# Knoevenagel condensation of 4,5- and 1,8-diazafluorenes

**DOI:** 10.3762/bjoc.22.62

**Published:** 2026-05-27

**Authors:** Darya S Cheshkina, Christina S Becker, Alina A Sonina, Maxim S Kazantsev

**Affiliations:** 1 N.N. Vorozhtsov Novosibirsk Institute of Organic Chemistry SB RAS, Lavrentieva 9, 630090 Novosibirsk, Russia

**Keywords:** acidic conditions, diazafluorene, diazafluorenylidene, Knoevenagel condensation, protonation

## Abstract

Diazafluorenylidenes are potential antitumor agents, ligands, and functional materials. However, there are currently only limited methods for their synthesis, which are complicated and typically based on modifications of 4,5-diazafluorenone. Herein, we systematically studied the Knoevenagel condensation of both 4,5- and 1,8-diazafluorenes with aromatic aldehydes and evaluated the diazafluorenes’ nature and solvent influence on the mechanism and reactivity. We also identified conditions allowing the preparation of both 4,5- and 1,8-diazafluorenylidenes in yields up to 85% minimizing complicated synthetic or experimental workup procedures. Additionally, the condensation of diazafluorenes with ketones resulted in unique di(pyridin-2-yl)methylene)-9*H*-diazafluorenes being electron-deficient ligands and functional materials.

## Introduction

Diazafluorenylidene derivatives were reported to be applied as antitumor agents [1–3], emitters and sensors [4–5]. They have also been extensively used as ligands yielding a wide library of transition-metal complexes [6–8], catalysts [9–10], light-driven molecular motors [11–12], nonlinear optical chromophores [13], and electrochromic materials [14]. Furthermore, diazafluorenylidenes may exhibit polymorphism, high solid-state emission, and decent charge carrier mobility which makes these molecules promising for use in organic optoelectronics [5,15–16].

Current synthetic approaches to diazafluorenylidenes are limited. The typical method for their synthesis involves coupling reactions of diazafluoren-9-one [4,6–7] or diazafluoren-9-diazomethane [17–18] with thiones yielding the products in moderate yields (20–70%). However, these routes require the preliminary synthesis of malodorous thiones and diazomethane derivatives from the corresponding ketones complicating the synthetic protocol. Therefore, the Knoevenagel condensation seemed to be an alternative and preferable approach. Previously reported syntheses of diazafluorenylidenes employed diazafluorenones as the carbonyl component which are reacted with an active methylene moiety like malononitrile or acetonitrile derivatives [14,19–20]. However, the use of diazafluorenes as the methylene component in condensation reactions remains underexplored. These reactions can be carried out under basic [13,21], acidic [3,5] or Lewis acid catalysis [15,22], but the corresponding literature examples are scarce, especially for 1,8-diazafluorene. We have previously reported the condensation of 4,5-diazafluorene (**1**) with aromatic aldehydes and dialdehydes under ammonium acetate catalysis in acetic acid giving the corresponding products in good yields [5,23]. Remarkably, the only condensation of 1,8-diazafluorene (**2**) was performed using a TiCl_4_/pyridine system. However, the reaction yield was very low (11%) [15]. The latter approach was also used [22] for the condensation of 4,5-diazafluorene with ketones being especially challenging due to sterical issues. Moreover, this approach requires the smelly solvent pyridine, moisture-sensitive environmentally aggressive reagent (TiCl_4_) and subsequent complicated treatment of the resulting reaction mixture making it inapplicable for large-scale high-efficiency production of functional materials. Therefore, analyzing the literature examples we assumed that 4,5- and 1,8-diazafluorenes may have different reactivity in the Knoevenagel condensation and the development of efficient and convenient synthetic routes to diazafluorenylidenes is of considerable importance.

Here, we systematically studied the Knoevenagel condensation of both 4,5- and 1,8-diazafluorenes with aromatic aldehydes and evaluated the influence of the diazafluorene nature and solvent on the reaction mechanisms and reactivity. The reactivity of diazafluorenes under basic conditions was demonstrated to be improved in protic solvents due to solvation of ion pairs by these solvents. We found that acidic conditions allow the preparation of both 4,5- and 1,8-diazafluorenylidenes in yields up to 85%. The basicity of the diazafluorenes was demonstrated to be different and the reaction of 1,8-diazafluorene is impeded because of electrostatic repulsion of the protonated tautomer and the iminium cation. Additionally, we performed the condensation of diazafluorenes with ketones yielding unique di(pyridin-2-yl)methylene)-9*H*-diazafluorenes.

## Results and Discussion

### Basic conditions

**Scheme 1:**
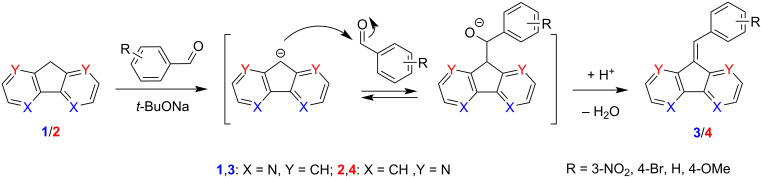
Knoevenagel reaction of **1** and **2** under basic conditions.

Following the typical protocol of the Knoevenagel condensation, the reactions of 4,5- and 1,8-diazafluorenes with several electron-donor and electron-acceptor-substituted benzaldehydes (viz*.* 4-methoxybenzaldehyde, 4-bromobenzaldehyde, 3-nitrobenzaldehyde, and unsubstituted benzaldehyde) were first carried out under basic conditions, using *t-*BuONa in DMF or *t-*BuOH (Scheme 1 and Table 1). Both diazafluorenes **1** and **2** reacted under such conditions, but only acceptor-substituted aldehydes were able to react in DMF, specifically 4-bromobenzaldehyde and 3-nitrobenzaldehyde. Though, the reaction in *tert*-butanol was successful for all studied substrates. These observations were attributed to solvation phenomena. Specifically, solvation by aprotic DMF having a high dipole moment promotes the formation of the [Na^+^–diazafluorenyl^−^] ion pair, whose anion displays insufficient nucleophilicity for interaction with weakly electrophilic aldehydes (e.g., benzaldehyde and 4-methoxybenzaldehyde). On the other hand, *tert*-butanol is effectively solvating ions via the formation of hydrogen bonds [24], thus promoting the dissociation of the ion pair and increasing the nucleophilicity of the diazafluorenyl anion, which becomes capable of interacting with less active aldehydes.

**Table 1:** Condensation conditions and yields for 4,5- (**1**) and 1,8-diazafluorenes (**2**).

Diazafluorene		**1**		**2**	

R		Product	Yield, % (solvent)	Product	Yield, % (solvent)

3-NO_2_	basic	**3a**	14^a^ (DMF)	**4a**	47^a^ (DMF)
			18^b^ (*t*-BuOH)		28^b^ (*t*-BuOH)
	acidic		38^c,d^ (AcOH)		0^c^ (AcOH)
					67^c,d^ (EtOH)
					50^c^ (toluene)

4-Br	basic	**3b**	28^a^ (DMF)	**4b**	32^a^ (DMF)
			28^b^ (*t*-BuOH)		45^b^ (*t*-BuOH)
	acidic		84^c,d^ (AcOH)		0^c^ (AcOH)
					60^c,d^ (EtOH)
					27^c^ (toluene)

H	basic	**3c**	0^a^ (DMF)	**4c**	0^a^ (DMF)
			34^b^ (*t*-BuOH)		57^b^ (*t*-BuOH)
	acidic		45^c,d^ (AcOH)		0^c^ (AcOH)
					85^c,d^ (EtOH)
					41^c^ (toluene)

4-OMe	basic	**3d**	0^a^ (DMF)	**4d**	0^a^ (DMF)
			29^b^ (*t*-BuOH)		27^b^ (*t*-BuOH)
	acidic		67^c,d^ (AcOH)		0^c^ (AcOH)
					39^c^ (EtOH)
					52^c^ (toluene)

Conditions: ^a^*t-*BuONa, solvent, rt, 2 h; ^b^*t-*BuONa, solvent, 30 °C,12 h; ^c^NH_4_OAc/AcOH, solvent, 110 °C, 12 h; ^d^the product precipitated directly from the reaction mixture.

### Acidic conditions

**Scheme 2:**
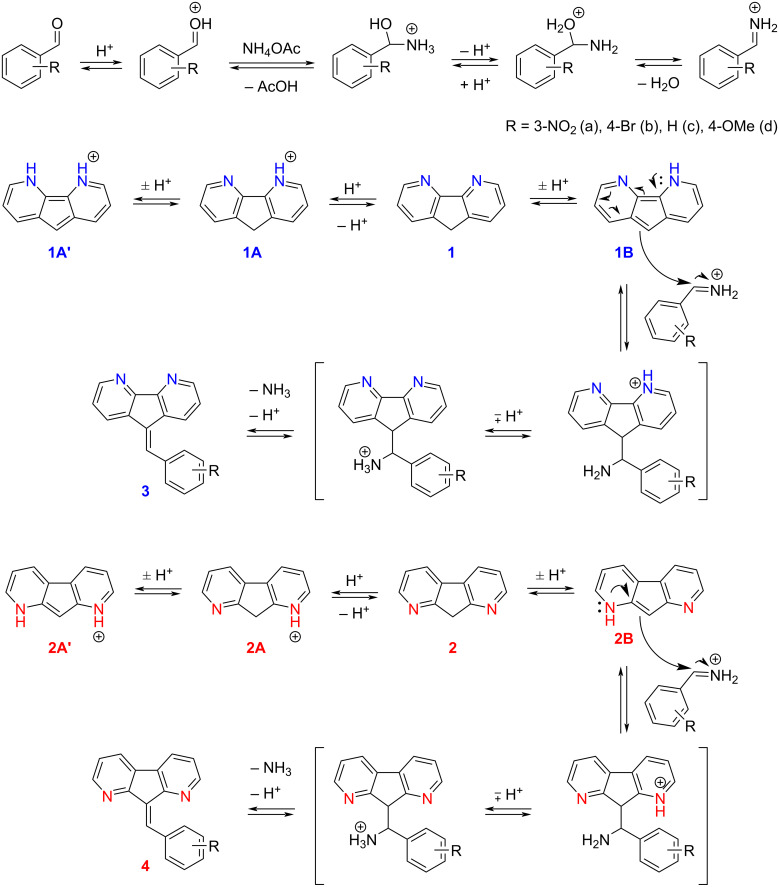
Knoevenagel reaction of **1** and **2** under acidic conditions. Proposed mechanism of condensation and competing protonation equilibrium.

As the reaction yields under basic conditions were moderate and the reactivity of aldehydes was insufficient it seemed reasonable to increase their activity by converting them into iminium cations through treatment with ammonium acetate in the presence of acetic acid (Scheme 2). Initially, the Knoevenagel condensation of **1** and **2** was carried out with the available set of aldehydes in glacial acetic acid as a solvent. The reactivity of diazafluorenes **1** and **2** under these conditions turned out to be different: while diazafluorene **1** readily reacted with all aldehydes studied delivering the products with good yields, diazafluorene **2** did not react at all – only starting compounds were quantitatively evaluated from all reactions (Scheme 2, Table 1). To rationalize this observation, we hypothesized that the protonation of nitrogens leads to electrostatic repulsion between the protonated diazafluorene and the iminium cation (vide infra). Therefore, we changed the solvent in the reactions of 1,8-diazafluorene (**2**) to ethanol or toluene. As a result, condensation products of **2** with various donor- and acceptor-substituted benzaldehydes were obtained in moderate to good yields in both solvents (Table 1). Note, the yields were generally higher in cases when the condensation product precipitated from the reaction mixture (see note d in Table 1), i.e., when the reaction became irreversible.

The proposed mechanism for the condensation reaction in the presence of ammonium acetate is shown in Scheme 2 [25]. Accordingly, the addition of diazafluorene to the iminium cation occurs via the methylene carbon of the corresponding enamine tautomeric form **1B** or **2B**. Thus, this process is limited by the concentration of this form which is expected to be higher for 1,8-diazafluorene (**2**) due to the activation by the neighboring nitrogen atoms via the mesomeric effect. On the other hand, diazafluorenes are potentially capable of reversible protonation by acetic acid, which is a competing process decreasing the equilibrium concentration of the enamine form (Scheme 2). Furthermore, an electrostatic repulsion between the protonated diazafluorene (**1A**/**1A’** or **2A**/**2A’**) and the iminium cation can be considered, and this effect should be stronger for 1,8-diazafluorene (**2**) because the nitrogen atoms are located near the reaction center.

To evaluate the possible protonation effect of 1,8- and 4,5-diazafluorenes we utilized ^1^H NMR experiments performed immediately after preparation and 6 hours later for solutions of **1** and **2** in pure CD_3_COOD and in CD_3_OD with a drop of CD_3_COOD. Remarkably, 4,5-diazafluorene did not undergo protonation in both experiments – only the signals of the neutral form of **1** were identified (see Supporting Information File 1, Figure S1). Contrary, both the neutral (**2**) and protonated (**2A’**) forms of 1,8-diazafluorene were identified in both solvents (see Supporting Information File 1, Figure S2) with relatively high ratio (**2** to **2A’** = 1:0.4 in CD_3_OD and 1:0.9 in CD_3_COOD). This equilibrium was reached immediately after the addition of the acid and demonstrated higher basicity of the nitrogens in 1,8-diazafluorene as compared with those of 4,5-diazafluorene. Therefore, the protonation (and corresponding repulsion impeding the condensation) effect plays the decisive role solely for compound **2**.

**Scheme 3:**
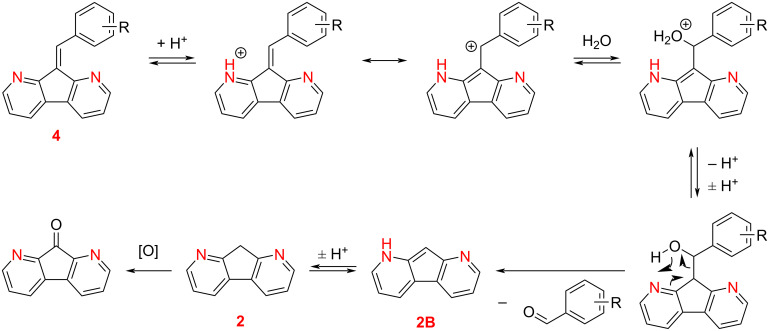
A proposed mechanism for the **4**-to-**2** degradation process.

Additionally, we evaluated the stability of the products in acidic media subjecting the condensation products **3b** and **4b** to heating in glacial acetic acid at 50 °C for 6 hours revealing that compound **3b** was stable whereas compound **4b** decomposed into the starting reagents (see the TLC data in Supporting Information File 1, Figure S3). Apparently, after the protonation and subsequent addition of a nucleophile (H_2_O) an intramolecular rearrangement facilitated by the proximity of a neighboring nitrogen atom (in position 1 or 8) occurred (see Scheme 3). Elimination of the aldehyde leads to the formation of 1,8-diazafluorene (**2**), which can be oxidized into 1,8-diazafluorenone by oxygen present in the reaction mixture. Therefore, the position of nitrogen atoms in diazafluorenes strongly affects their reactivity in acidic media via protonation, electrostatic repulsion, and stability of the condensation products. Therefore, the equilibrium in the reaction of fluorene **2** carried out in acetic acid is shifted toward the starting compounds. Hence, the Knoevenagel condensation of 1,8-diazafluorene (**2**) should be performed using ammonium acetate, catalytic amounts of acetic acid in a non-acidic solvent.

Going beyond the mechanistic and reactivity insights we applied this approach for the synthesis of 1,4-bis((9*H*-(1,8-diazafluoren)-9-ylidene)methyl)phenylene (**4е**, Scheme S1 in Supporting Information File 1) which resulted in 65% yield significantly surpassing the previously reported 11% using the TiCl_4_ system [15]. The method shown here, in contrast to condensation using TiCl_4_ and pyridine, is cheaper, easier to apply, and the pure target product simply precipitated from the reaction mixture instead of extraction and purification by column chromatography.

### Di(pyridin-2-yl)methylene)-9*H*-diazafluorenes

Furthermore, since ketones are generally considered to be less reactive than aldehydes and may also suffer from steric hindrance during the reaction to further demonstrate the practical potential of Knoevenagel condensation we implemented our acidic conditions and involved **1** and **2** into reaction with di(pyrid-2-yl)ketone with subsequent evaluation of the products’ structure, physicochemical properties and coordination behavior (for 4,5-derivative).

**Scheme 4:**
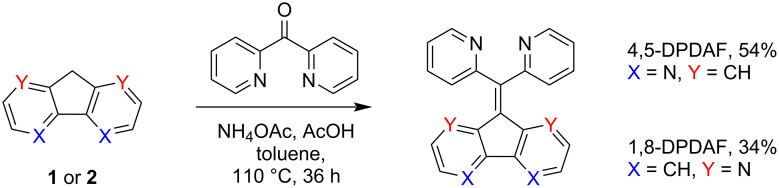
Condensation of 4,5- (**1**) and 1,8-diazafluorene (**2**) with di(pyrid-2-yl)ketone. Reaction yields are given in parentheses.

**Figure 1:**
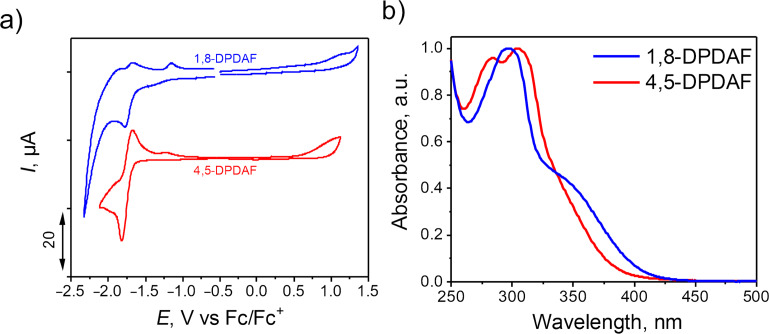
a) Cyclic voltammograms of compounds 4,5-DPDAF and 1,8-DPDAF in CH_2_Cl_2_ solution and b) optical absorption spectra of 4,5-DPDAF and 1,8-DPDAF in THF.

**Figure 2:**
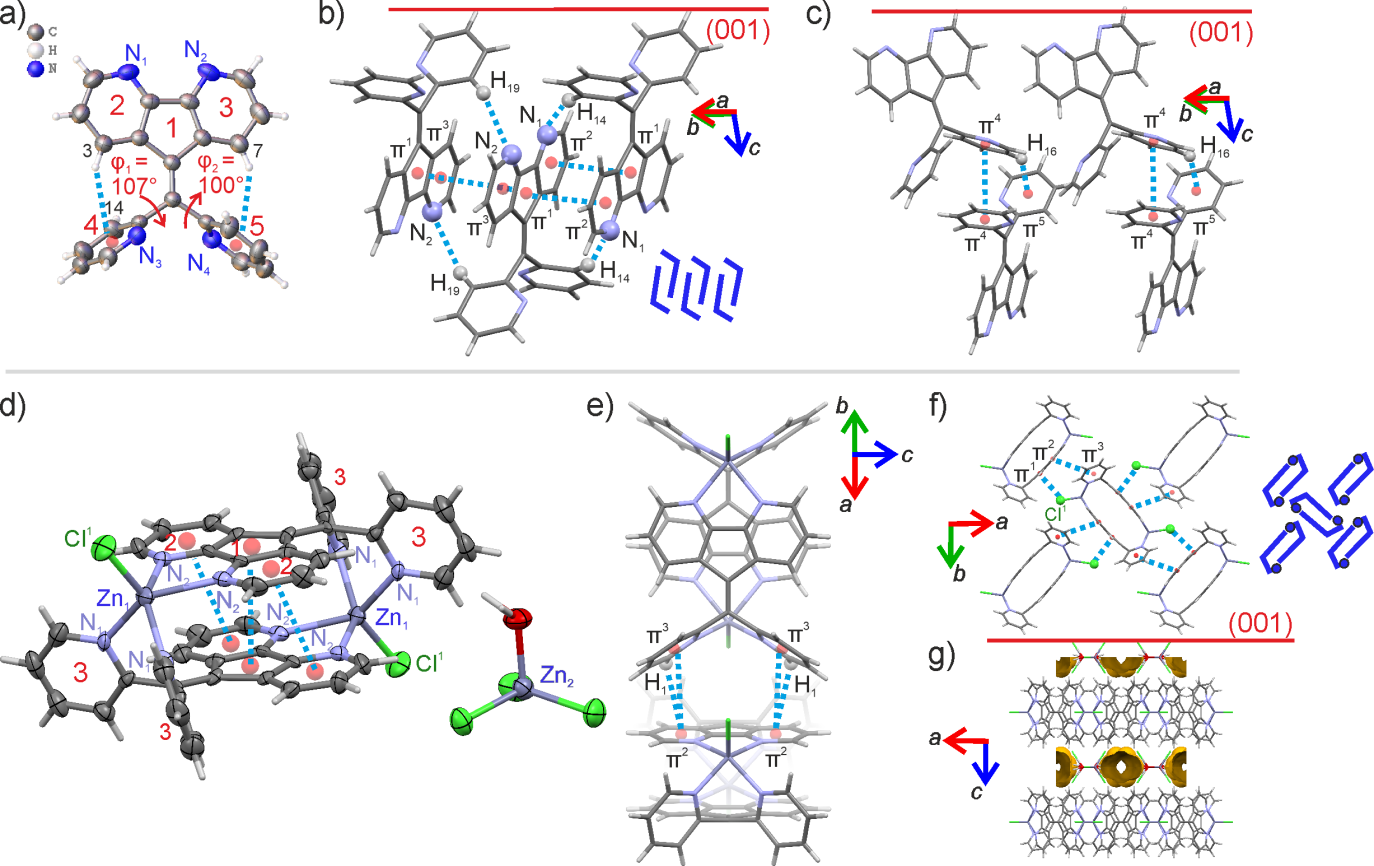
Molecular structure, atom and cycle numbering of 4,5-DPDAF (a) and Zn-4,5-DPDAF (d) with anisotropic displacement ellipsoids drawn at a 50% probability level; crystal structure fragments of 4,5-DPDAF with π-stacking (b) and C–H···π interactions (c); crystal structure fragments of Zn-4,5-DPDAF with π-stacking between dimers (e), their top view (f) and the layered packing of the compound depicting the voids (g). Dashed blue lines represent noncovalent interactions. The arrows indicate the orientation of crystallographic axes. The red line shows the (001) plane. The schematic packing motifs are shown in blue, whereas linking [ZnCl]^+^ moieties are illustrated by blue dots. Voids are shown as brown contact surfaces created using a probe radius of 1.2 Å.

Both 4,5- and 1,8-diazafluorenes reacted with di(pyrid-2-yl)ketone forming di(pyridin-2-yl)methylene)-9*H*-4,5-diazafluorene (4,5-DPDAF) and 9-(di(pyridin-2-yl)methylene)-9*H*-1,8-diazafluorene (1,8-DPDAF) in 54% and 34% yields, respectively (Scheme 4). Ammonium acetate and acetic acid in toluene were chosen as conditions providing good solubility of reagents. To the best of our knowledge these reactions are the first examples of a titanium-free condensation of diazafluorenes with ketones. The obtained diazafluorenylidenes were characterized by NMR, HRMS, elemental analysis, cyclic voltammetry (CV), UV–vis spectroscopy (Figure 1) and X-ray data (Figure 2 and Figure S4 in Supporting Information File 1).

Figure 1a demonstrates the cyclic voltammograms of DPDAFs in CH_2_Cl_2_ solution. Both compounds demonstrated pronounced quasi-reversible reduction waves with half-wave potentials *E*_1/2_ = −1.74 and −1.68 V (vs Fc/Fc^+^) for compounds 4,5-DPDAF and 1,8-DPDAF, respectively. The LUMO energies estimated using the onset reduction potentials were −3.12 eV and −3.15 eV for 4,5-DPDAF and 1,8-DPDAF, respectively, indicating a very similar electron-accepting effect of 4,5- and 1,8-diazafluorenes. Figure 1b demonstrates the optical absorption spectra of 4,5-DPDAF and 1,8-DPDAF in THF solution. The spectra have similar absorption maxima at 304 nm and 297 nm for 4,5-DPDAF and 1,8-DPDAF, respectively. However, the absorption spectrum edge demonstrated a bathochromic shift for 1,8-DPDAF (400 nm) as compared to that of 4,5-DPDAF (380 nm) what we assigned to the slightly higher planarity for 1,8-DPDAF due to its lower sterical hindrance. Based on the energies of absorption edges we estimated *E*_g_ to be 3.3 eV and 3.1 eV for 4,5-DPDAF and 1,8-DPDAF, respectively. Accordingly, the HOMO energies were −6.38 eV and −6.25 eV for 4,5-DPDAF and 1,8-DPDAF being in accordance with the high electron affinity of diazafluorenes.

As a next step we solved the crystal structures of both compounds. Here we discuss the structure of 4,5-DPDAF and the data for compound 1,8-DPDAF is presented in Supporting Information File 1, note S1. Compound 4,5-DPDAF crystallizes as yellow needles. The conformation of the pyridine fragments is non-planar relative to the diazafluorene fragment due to sterical repulsion of the pyridine moieties and additionally due to C–H···π intramolecular interactions: φ_1_ = 106.8(2)° (N_3_–C_13_–C_12_–C_5_) and φ_2_ = 99.6(2)° (N_4_–C_18_–C_12_–C_5_) (Figure 2a). The molecules of 4,5-DPDAF are packed into stacks due to π-stacking interactions between diazafluorenes which are stabilized by C–H···N interactions between pyridine and diazafluorene fragments along (a + b) (Figure 2b). The stacks of molecules are linked together via π···π and C–H···π interactions between pyridine fragments (Figure 2c). To further evaluate the complexation behavior of 4,5-DPDAF we co-crystallized the compound with ZnCl_2_ resulting in a unique complex (Zn**–**4,5-DPDAF) where Zn is four-coordinated to the nitrogens of the diazafluorene and pyridine rings forming a positively charged cyclic dimer (Figure 2d). The dimers are arranged in a herringbone-type structure via C–H···π, C–Cl···π and π-stacking interactions between diazafluorene and pyridine (Figure 2f). Layers of dimers alternate with layers of ZnCl_3_(H_2_O)^−^ anions containing free solvent accessible volume 284 Å^3^ with removed disordered water molecules (Figure 2g). Figure 2 also shows a schematic representation of the packing difference of the compounds in blue, showing that the dimers in 4,5-DPDAF molecules form stacks, while in the complex the dimers with Zn form a herringbone packing. The complex Zn–4,5-DPDAF represents a unique cyclic dimer structure and highlights a high potential of diazafluorenylidenes for coordination chemistry and materials science.

## Conclusion

In summary, we elaborated efficient Knoevenagel condensation conditions giving 4,5- and 1,8-diazafluorenylidenes in good yields avoiding complicated synthetic or experimental workup procedures. 1,8-Diazafluorene readily condenses with aromatic aldehydes using NH_4_OAc and AcOH in a non-acidic solvent whereas the reaction for 4,5-diazafluorene may be carried out in pure acetic acid. According to NMR studies, 4,5-diazafluorene has a lower nitrogen basicity as compared to 1,8-diazafluorene. This basicity plays a key role in the reaction of 1,8-diazafluorene condensation because of electrostatic repulsion of the protonated tautomer and the iminium cation. Additionally, the nitrogen positions in 1,8-diazafluorenylidenes facilitate their decomposition in acidic media. Moreover, the reactivity of diazafluorenes under basic conditions also demonstrated to be improved via solvation of the ion pair by a protic solvent. Finally, the unique di(pyridin-2-yl)methylene)-9*H*-diazafluorenes were obtained by condensation of 4,5- and 1,8-diazafluorenes with di(pyrid-2-yl)ketone highlighting the high synthetic potential of the Knoevenagel reaction on the way toward novel electron-deficient ligands and functional materials.

## Experimental

### General

4,5-Diazafluorene (**1**) was synthesized in two steps from 1,10-phenanthroline monohydrate according to literature using a slightly modified procedure [21]. 1,8-Diazafluorene (**2**) was obtained using the method described in [15]. All reagents and solvents were purchased from commercial sources and used without additional purification. Reaction mixtures were monitored by TLC using Macherey-Nagel pre-coated TLC-sheets Alugram Xtra SIL G/UV254. For column chromatography Macherey-Nagel Kieselgel 60 was used. NMR spectra of products were recorded with Bruker AV 300, Bruker AV 400, and Bruker DRX 500 spectrometers in CDCl_3_ or (CD_3_)_2_CO. NMR samples of the solutions of 4,5- and 1,8-diazafluorenes (6 mg/0.5 mL) for the protonation study were prepared by dissolving **1** and **2** in either pure CD_3_COOD or CD_3_OD containing 0.005 mL of CD_3_COOD. Subsequently, ^1^H NMR spectra were recorded with a Bruker AV 400 immediately after sample preparation and 6 hours later. HRMS were obtained with a Thermo Scientific Double Focusing System (DFS) high-resolution mass spectrometer. Samples were introduced into the mass spectrometer by direct inlet. Electron ionization with 70 eV energy was used. Measurements of the exact masses of molecular ions were performed with respect to the standard lines of perfluorokerosene (PFK). Elemental analyses were performed for C, H, and N with a CHN-Analyzer Euro EA 300. Melting points were determined with a Thermosystem FP 900 instrument (Mettler Toledo). UV–vis spectra were recorded in diluted (10^−5^ M) THF in 1 × 1 cm quartz cuvettes using a Varian Cary 5000 UV-VIS-NIR spectrometer. Cyclic voltammetry measurements were performed in CH_2_Cl_2_ solution by computer-controlled P-20x potentiostat (SmartStat, Russia) in combination with a three-electrode cell (Gamry); 0.1 M tetrabutylammonium hexafluorophosphate was used as supporting electrolyte. Pt, Pt wire, and Ag/AgCl were used as working, counter, and reference electrodes, respectively. The measurements were standardized by measuring the red/ox potential of ferrocene after each compound analysis. All potentials are reported vs Fc/Fc^+^ red/ox couple. LUMO energies were estimated using the onset red/ox potentials according to the equation: *E*_lumo_= −(*E*_red_^onset^ + 4.8) (eV)

**X-ray study.** Neat compounds were crystallized by a slow vapor diffusion method from the CHCl_3_/hexane (4,5-DPDAF) and toluene/iPrOH (1,8-DPDAF) systems. The crystal growth time was about 3 days. The crystals of Zn-4,5-DPDAF (Zn**–**4,5-DPDAF) were grown by layering a near-saturated ZnCl_2_ solution in CH_3_OH over the CHCl_3_ solution of 4,5-DPDAF. The crystal growth time was about 2 weeks. Single-crystal X-ray diffraction experiments were performed at 296(2) K using a Bruker KAPPA APEX II and Tongda TD-5000 diffractometer with Mo Kα radiation (λ = 0.71073 Å), equipped with a hybrid photon-counting detector (Supporting Information File 1, Table S1). Integration and scaling of the intensity data for Bruker were accomplished with SAINT [26]. Absorption corrections were applied using SADABS [27]. The obtained diffraction data from Tongda were converted into a standard ESPERANTO format [28]. Data reduction, scaling, and absorption correction were carried out using the CrysAlis PRO program package (v. 1.171.42.49). Structure was determined and refinement was performed using SHELXT [29] and SHELXL [30] programs with Olex2 (v. 1.5) [31]. All non-hydrogen atoms were refined anisotropically. The positions of hydrogen atoms were treated using constrained refinement using a riding model [Uiso (H) = 1.2 Ueq (C)]. Structure validation was carried out using CheckCIF service [32]. The structural data of crystals were deposited as CIF files at the Cambridge Crystallographic Database (CCDC No. 2517007 (4,5-DPDAF), 2517008 (1,8-DPDAF), 2517006 (Zn-4,5-DPDAF)) and can be downloaded freely from the following site: https://www.ccdc.cam.ac.uk. Mercury [33], Olex2, and PLATON [32] were used for visualization and analysis of the crystal structure. The free solvent accessible volume in compounds Zn-4,5-DPDAF and 1,8-DPDAF derived from Olex2 “Solvent Mask” routine (PLATON/SQUEEZE procedure) in analysis was found to be 11% (284 Å^3^, 100 electrons) and 23% (984 Å^3^, 136 electrons) accordingly. This volume is occupied by highly disordered water molecules (as evidenced from residual electron density peaks) that could not be modeled as a set of discrete atomic sites.

### Synthesis

#### General method (A1) for the condensation in acetic acid

In a manner analogous to [5]. A mixture of diazafluorene (30.0 mg, 0.18 mmol), ammonium acetate (60.5 mg, 0.79 mmol), and aldehyde (0.20 mmol) in glacial acetic acid (5 mL) was heated to 110 °C with stirring for 12 h. The resultant solution was cooled to room temperature and neutralized with aqueous ammonia solution (25%) to pH 6. The precipitate of a product was filtered off and washed sequentially with water, methanol, hexane, then dried in air.

#### General method (A2) for the condensation in other acidic conditions

A mixture of diazafluorene (30.0 mg, 0.18 mmol), ammonium acetate (60.5 mg, 0.79 mmol), and aldehyde (0.20 mmol), glacial acetic acid (0.02 mL, 0.36 mmol), ethanol or toluene (5 mL) was heated to 110 °C with stirring for 12 h. The resultant solution was cooled to room temperature and neutralized with aqueous ammonia solution (25%) to pH 6. The precipitated compounds (see Table 1) were isolated by filtration followed by washing sequentially with portions of water, methanol, hexane, and dried in air. In the case of absence of any precipitate, the solutions were extracted with chloroform, dried over anhydrous MgSO_4_ and concentrated under reduced pressure. The products were purified either by column chromatography on silica gel (eluent ethyl acetate) or by addition of diethyl ether to the residue after evaporation followed by filtration of the resulting precipitate, which was then washed with diethyl ether, and dried in air.

#### General method (B1) for the condensation in basic conditions

In a manner analogous to [34]. A solution of diazafluorene (30.0 mg, 0.18 mmol) in DMF (5 mL) was purged with argon for 20 minutes. Then, *t*-BuONa (20.6 mg, 0.21 mmol) was added, and the flask was sealed with a septum. The mixture was stirred vigorously for 10 min, and then a solution of an aldehyde (0.20 mmol) in anhydrous DMF (2 mL) was added dropwise. The reaction mixture was stirred at room temperature for 2 h and then poured into water and extracted with chloroform. The extract was dried over MgSO_4_ and concentrated under reduced pressure. The products were purified by column chromatography on silica gel using ethyl acetate as eluent.

#### General method (B2) for the condensation in basic conditions

A solution of diazafluorene (30.0 mg, 0.18 mmol) in *t*-BuOH (5 mL) was purged with argon for 20 minutes. Then, *t*-BuONa (20.6 mg, 0.21 mmol) was added, and the flask was sealed with a septum. The mixture was stirred vigorously for 10 min, and then a solution of an aldehyde (0.20 mmol) in *t*-BuOH (2 mL) was added dropwise. The reaction mixture was heated to 30 °C and stirred at this temperature for 12 h, then poured into water and extracted with chloroform. The extract was dried over MgSO_4_ and concentrated under reduced pressure. The products were purified by column chromatography on silica gel using ethyl acetate as eluent.

The data on the yield, melting point, elemental analysis and spectral characteristics of each compound are provided in Supporting Information File 1 (section 1 and Figures S5–S34).

#### Synthesis of 1,4-bis((9*H*-(1,8-diazafluoren)-9-ylidene)methyl)phenylene (**4e**)

A mixture of 1,8-diazafluorene (30.0 mg, 0.18 mmol), terephthalaldehyde (10.8 mg, 0.08 mmol), ammonium acetate (55.4 mg, 0.72 mmol) and glacial acetic acid (0.02 mL, 0.36 mmol) in toluene (5 mL) was heated to 110 °C with stirring for 12 h. The resultant solution was cooled to room temperature and neutralized with aqueous ammonia solution (25%) to pH 6. The resulting precipitate was filtered, washed successively with water, methanol, hexane and dried in air. Yield: 22.7 mg, 65%. The NMR spectrum corresponds to that described in [15].

## Supporting Information

File 1 Analytical data, protonation data, thin-layer chromatography data, X-ray data, spectra of synthesized compounds.

File 2 Crystallographic information files for compounds 1,8-DPDAF, 4,5-DPDAF and Zn–4,5-DPDAF.

## Data Availability

All data that supports the findings of this study is available in the published article and/or the supporting information of this article.
