# Supplementary material for: Knoevenagel condensation of 4,5- and 1,8-diazafluorenes
Source: Beilstein J Org Chem. 2026 May 27;22:803–12. doi: 10.3762/bjoc.22.62 (PMC13224056; doi:10.3762/bjoc.22.62)
Supplement: File 1 — Analytical data, protonation data, thin-layer chromatography data, X-ray data, spectra of synthesized compounds. [file Beilstein_J_Org_Chem-22-803-s001.pdf]

BEILSTEIN JOURNAL OF ORGANIC CHEMISTRY

Supporting Information

for

Knoevenagel condensation of 4,5- and 1,8-diazafluorenes

Darya S. Cheshkina, Christina S. Becker, Alina A. Sonina and Maxim S. Kazantsev

Beilstein J. Org. Chem. **2026,** *22,* 803–812. doi:10.3762/bjoc.22.62

Analytical data, protonation data, thin-layer chromatography data, X-ray data, spectra of synthesized compounds

License and Terms: This is a supporting information file under the terms of the Creative Commons Attribution License (https://creativecommons.org/licenses/by/4.0). Please note that the reuse, redistribution and reproduction in particular requires that the author(s) and source are credited and that individual graphics may be subject to special legal provisions.
The license is subject to the *Beilstein Journal of Organic Chemistry* terms and conditions: (https://www.beilstein-journals.org/bjoc/terms)

1. Analytical data

9-(3-Nitrobenzylidene)-9*H*-4,5-diazafluorene (3a).

Yields: 20.4 mg, 38% (method A1); 7.5 mg, 14% (method B1), 15.1 mg, 18% (method B2); mp 208.1–209.9 °C; δ_H (CDCl$_3$, 500.13 MHz): 8.76 (d, J = 3.3 Hz, 1H), 8.67 (d, J = 3.3 Hz, 1H), 8.43 (s, 1H), 8.31 (d, J = 7.5 Hz, 1H), 8.12 (d, J = 7.5 Hz, 1H), 7.92 (d, J = 7.2 Hz, 1H), 7.74 (s, 1H), 7.72–7.67 (m, 2H), 7.37 (dd, ^1J = 7.5 Hz, ^2J = 4.9 Hz, 1H), 7.10 (dd, ^1J = 7.5 Hz, ^2J = 4.9 Hz, 1H); δ_C (CDCl$_3$, 125.76 MHz): 158.7, 156.8, 151.1, 151.1, 148.6, 137.5, 135.4, 133.4, 133.2, 131.2, 130.6, 130.1, 128.0, 127.8, 124.2, 123.7, 123.3, 122.8; HRMS found: $[M]^{+\cdot}$ 301.0847, $C_{18}H_{11}N_3O_2$ requires $[M]^{+\cdot}$ 301.0846; Elem. Analysis. Found: C, 71.68; H, 3.75; N, 13.72% for $C_{18}H_{11}N_3O_2$, requires: C, 71.75; H, 3.68; N, 13.95%.

9-(4-Bromobenzylidene)-9*H*-4,5-diazafluorene (3b).

Yields: 50.2 mg, 84% (method A1); 16.7 mg, 28% (method B1); 16.7 mg, 28% (method B2); mp 209.2–210.4 °C; δ_H (CDCl$_3$, 500.13 MHz): 8.73 (d, J = 3.9 Hz, 1H), 8.66 (d, J = 3.6 Hz, 1H), 8.09 (d, J = 7.2 Hz, 1H), 7.83 (d, J = 7.2 Hz, 1H), 7.68 (s, 1H), 7.62 (d, J = 7.9 Hz, 2H), 7.44 (d, J = 7.9 Hz, 2H), 7.34 (dd, ^1J = 3.9 Hz, ^2J = 7.2 Hz, 1H), 7.11 (dd, ^1J = 3.6 Hz, ^2J = 7.2 Hz, 1H); δ_C (CDCl$_3$, 125.76 MHz): 158.4, 156.5, 150.7, 134.7, 133.7, 132.1, 131.5, 131.4, 131.0, 129.9, 127.8, 123.3, 123.1, 122.6; HRMS found: $[M]^{+\cdot}$ 334.0099, $C_{18}H_{11}N_2Br$ requires $[M]^{+\cdot}$ 334.0100; Elem. Analysis. Found: C, 64.52; H, 3.34; Br, 23.92; N, 8.22% for $C_{18}H_{11}N_2Br$, requires: C, 64.50; H, 3.31; Br, 23.84; N, 8.36%.

9-Benzylidene-9*H*-4,5-diazafluorene (3c).

Yields: 20.6 mg, 45% (method A1), 15.5 mg, 34% (method B2); mp 155.8–158.1 °C; δ_H (CDCl$_3$, 500.13 MHz): 8.74 (d, J = 2.9 Hz, 1H), 8.65 (d, J = 2.8 Hz, 1H), 8.12 (d, J = 7.8 Hz, 1H), 7.87 (d, J = 7.7 Hz, 1H), 7.80 (s, 1H), 7.57 (d, J = 6.8 Hz, 2H), 7.51–7.48 (m, 2H), 7.46 (d, J = 7.1 Hz, 1H), 7.35 (dd, ^1J = 2.9 Hz, ^2J = 7.8 Hz, 1H), 7.09 (dd, ^1J = 2.8 Hz, ^2J = 7.7 Hz, 1H); δ_C (CDCl$_3$, 125.76 MHz): 158.3, 156.4, 150.5, 135.8, 133.9, 131.5, 131.5, 131.3, 130.9, 129.4, 129.0, 128.9, 127.7, 123.1, 122.6; the NMR spectrum corresponds to that described in [1]; HRMS found: $[M-1]^{+\cdot}$ 255.0916, $C_{18}H_{11}N_2$ requires $[M-1]^{+\cdot}$ 255.0917; Elem. Analysis. Found: C, 84.12; H, 4.94; N, 10.94% for $C_{18}H_{12}N_2$, requires: C, 84.35; H, 4.72; N, 10.93%.

9-(4-Methoxybenzylidene)-9*H*-4,5-diazafluorene (3d).

Yields: 34.2 mg, 67% (method A1); 14.8 mg, 29% (method B2); mp 235.5–237.3 °C; δ_H (CDCl$_3$, 300.13 MHz): 8.71 (dd, ^1J = 1.2 Hz, ^2J = 4.9 Hz, 1H), 8.65 (dd, ^1J = 1.2 Hz, ^2J = 4.9 Hz, 1H), 8.08 (dd, ^1J = 1.2 Hz, ^2J = 7.8 Hz, 1H), 8.03 (dd, ^1J = 1.2 Hz, ^2J = 8.1 Hz, 1H), 7.73 (s, 1H), 7.53 (d, J =

8.6 Hz, 2H), 7.32 (dd, ^1J = 4.9 Hz, ^2J = 7.8 Hz, 1H), 7.11 (dd, ^1J = 4.9 Hz, ^2J = 8.1 Hz, 1H), 7.01 (d, J = 8.6 Hz, 2H), 3.89 (s, 3H); δ_C ($CDCl_3$, 75.47 MHz): 160.3, 158.1, 156.1, 150.2, 150.1, 134.1, 131.6, 131.3, 131.2, 131.2, 129.6, 128.0, 127.5, 122.9, 122.5, 114.3, 55.5; HRMS found: $[M]^+$ 286.1103, $C_{19}H_{14}N_2O$ requires $[M]^+$ 286.1101; Elem. Analysis. Found: C, 80.02; H, 4.91; N, 9.71% for $C_{19}H_{14}N_2O$, requires: C, 79.70; H, 4.93; N, 9.78%.

9-(3-Nitrobenzylidene)-9*H*-1,8-diazafluorene (4a).

Yields: 36.0 mg, 67% (method A2, ethanol); 26.9 mg, 50% (method A2, toluene); 25.3 mg, 47% (method B1); 15.1 mg, 28% (method B2); mp 184.7–186.0 °C; δ_H ($CDCl_3$, 500.13 MHz): 10.03 (s, 1H), 8.70–8.67 (m, 2H), 8.59 (d, J = 4.3 Hz, 1H), 8.28 (s, 1H), 8.26 (d, J = 8.8 Hz, 1H), 8.04 (d, J = 7.3 Hz, 1H), 7.98 (d, J = 7.3 Hz, 1H), 7.64 (dd, ^1J = 7.8 Hz, ^2J = 7.8 Hz, 1H),, 7.35–7.31 (m, 2H); δ_C ($CDCl_3$, 125.76 MHz): 157.0, 155.0, 148.8, 148.4, 138.7, 136.7, 134.9, 132.8, 132.3, 130.3, 129.2, 128.0, 127.9, 124.4, 123.3; HRMS found: $[M-1]^{+\cdot}$ 300.0767, $C_{18}H_{10}N_3O_2$ requires $[M-1]^{+\cdot}$ 300.0768; Elem. Analysis. Found: 71.72; H, 3.64; N, 13.89% for $C_{18}H_{11}N_3O_2$, requires: C, 71.75; H, 3.68; N, 13.95%.

9-(4-Bromobenzylidene)-9*H*-1,8-diazafluorene (4b).

Yields: 35.9 mg, 60% (method A2, ethanol); 16.2 mg, 27% (method A2, toluene); 19.1 mg, 32% (method B1); 26.8 mg, 45% (method B2); mp 162.3–163.6 °C; δ_H ($CDCl_3$, 500.13 MHz): 8.64 (d, J = 4.3 Hz, 1H), 8.60 (d, J = 8.2 Hz, 2H), 8.56 (d, J = 4.3 Hz, 1H), 8.25 (s, 1H), 8.02 (d, J = 7.5 Hz, 1H), 7.98 (d, J = 7.5 Hz, 1H), 7.63 (d, J = 8.2 Hz, 2H), 7.32–7.28 (m, 2H); δ_C ($CDCl_3$, 125.76 MHz): 157.5, 155.4, 148.7, 148.2, 134.8, 134.4, 134.2, 132.9, 132.4, 131.6, 129.8, 127.7, 127.6, 124.9, 122.7, 122.6; HRMS found: $[M-1]^{+\cdot}$ 333.0016, $C_{18}H_{10}N_2Br$ requires $[M-1]^{+\cdot}$ 333.0022; Elem. Analysis. Found: 64.57; H, 3.35; Br, 23.96; N, 8.12% for $C_{18}H_{10}N_2Br$, requires: C, 64.50; H, 3.31; Br, 23.84; N, 8.36%.

9-Benzylidene-9*H*-1,8-diazafluorene (4c).

Yields: 38.9 mg, 85% (method A2, ethanol); 18.7 mg, 41% (method A2, toluene); 26.1 mg, 57% (method B2); mp 112.1–113.9 °C; δ_H ($CDCl_3$, 400.13 MHz): 8.72 (d, J = 7.5 Hz, 2H), 8.67 (d, J = 4.1 Hz, 1H), 8.60 (d, J = 4.2 Hz, 1H), 8.36 (s, 1H), 8.04 (d, J = 7.5 Hz, 1H), 7.99 (d, J = 7.6 Hz, 1H), 7.54–7.50 (m, 2H), 7.47–7.43 (m, 1H), 7.32–7.28 (m, 1H); δ_C ($CDCl_3$, 75.47 MHz):157.7, 155.6, 148.6, 148.3, 136.0, 135.2, 133.3, 132.2, 130.4, 129.7, 128.4, 127.7, 127.6, 122.6, 122.5; HRMS found: $[M-1]^{+\cdot}$ 255.0913, $C_{19}H_{11}N_2$ requires $[M-1]^{+\cdot}$ 255.0917; Elem. Analysis. Found: C, 84.42; H, 4.79; N, 10.79% for $C_{18}H_{12}N_2$, requires: C, 84.35; H, 4.72; N, 10.93%.

9-(4-Methoxybenzylidene)-9*H*-1,8-diazafluorene (4d).

Yields: 19.9 mg, 39% (method A2, ethanol); 26.6 mg, 52% (method A2, toluene); 13.8 mg, 27% (method B2); mp 155.9 °C with subsequent decomposition; δ_H ($CDCl_3$, 300.13 MHz): 8.83 (d, J = 8.8 Hz, 2H), 8.69 (d, J = 4.8 Hz, 1H), 8.59 (d, J = 4.8 Hz, 1H), 8.33 (s, 1H), 8.06 (dd, ^1J = 0.9 Hz, ^2J = 7.6 Hz, 1H), 8.00 (dd, ^1J = 0.9 Hz, ^2J = 7.7 Hz, 1H), 7.32–7.28 (m, 2H), 7.05 (d, J = 8.8 Hz, 2H), 3.91 (s, 3H); δ_C ($CDCl_3$, 125.76 MHz):161.5, 157.9, 155.6, 148.4, 148.0, 136.0, 135.7, 131.7, 129.7, 129.1, 128.3, 127.6, 127.6, 122.0, 121.9, 114.0, 55.5; HRMS found: $[M{-}1]^{+\cdot}$ 285.1023, $C_{19}H_{13}O_1N_2$ requires $[M{-}1]^{+\cdot}$ 285.1022; Elem. Analysis. Found: C, 79.81; H, 4.89; N, 9.69% for $C_{19}H_{14}N_2O$, requires: C, 79.70; H, 4.93; N, 9.78%.

9-(Di(pyridin-2-yl)methylene)-9*H*-4,5-diazafluorene (4,5-DPDAF).

Yield: 32.2 mg, 54% (method A2, toluene); mp 252.9–255.1°C; δ_H ($CDCl_3$, 500.13 MHz): 8.77 (d, J = 3.4 Hz, 2H), 8.60 (d, J = 3.4 Hz, 2H), 7.82 (dd, 1J = 7.3 Hz, 2J = 7.3 Hz, 2H), 7.57 (d, J = 7.3 Hz, 2H), 7.41 (dd, ^1J = 4.9 Hz, ^2J = 7.3 Hz, 2H), 6.97 (dd, ^1J = 4.9 Hz, ^2J = 7.3 Hz, 2H), 7.57 (d, J = 7.3 Hz, 2H); δ_C ($CDCl_3$, 125.76 MHz): 158.2, 158.0, 150.5, 144.6, 137.3, 132.6, 132.0, 131.8, 125.5, 123.8, 122.6; HRMS found: $[M{-}1]^{+\cdot}$ 333.1138, $C_{22}H_{13}N_4$ requires $[M{-}1]^{+\cdot}$ 333.1135; Elem. Analysis. Found: C, 79.25; H, 4.18; N, 16.57% for $C_{22}H_{14}N_4$, requires: C, 79.02; H, 4.22; N, 16.76%.

9-(Di(pyridin-2-yl)methylene)-9*H*-1,8-diazafluorene (1,8-DPDAF).

Yield: 20.3 mg, 34% (method A2, toluene); mp 189.4 °C with subsequent decomposition; δ_H ($(CD_3)_2CO$, 500.13 MHz): 8.53 (ddd, ^1J = 4.9 Hz, ^2J = 1.7 Hz, ^3J = 0.9 Hz, 2H), 8.19 (dd, ^1J = 7.7 Hz, ^2J = 1.7 Hz, 2H), 8.16 (dd, ^1J = 4.8 Hz, ^2J = 1.5 Hz, 2H), 7.75 (ddd, ^1J = 7.7 Hz, ^2J = 7.7 Hz, ^3J = 1.8 Hz, 2H), 7.64 (d, J = 7.8 Hz, 2H), 7.30 (ddd, ^1J = 7.5 Hz, ^2J = 5.0 Hz, ^3J = 1.2 Hz, 2H), 7.27 (dd, ^1J = 7.7 Hz, ^2J = 4.9 Hz, 2H); δ_C ($(CD_3)_2CO$, 125.76 MHz): 159.9, 157.1, 149.6, 148.8, 135.7, 133.6, 133.6, 132.4, 128.5, 127.3, 123.8, 123.2; HRMS found: $[M{-}1]^{+\cdot}$ 333.1133, $C_{22}H_{13}N_4$ requires $[M{-}1]^{+\cdot}$ 333.1135; Elem. Analysis. Found: C, 79.15; H, 4.25; N, 16.60% for $C_{22}H_{14}N_4$, requires: C, 79.02; H, 4.22; N, 16.76%.

2. Protonation data

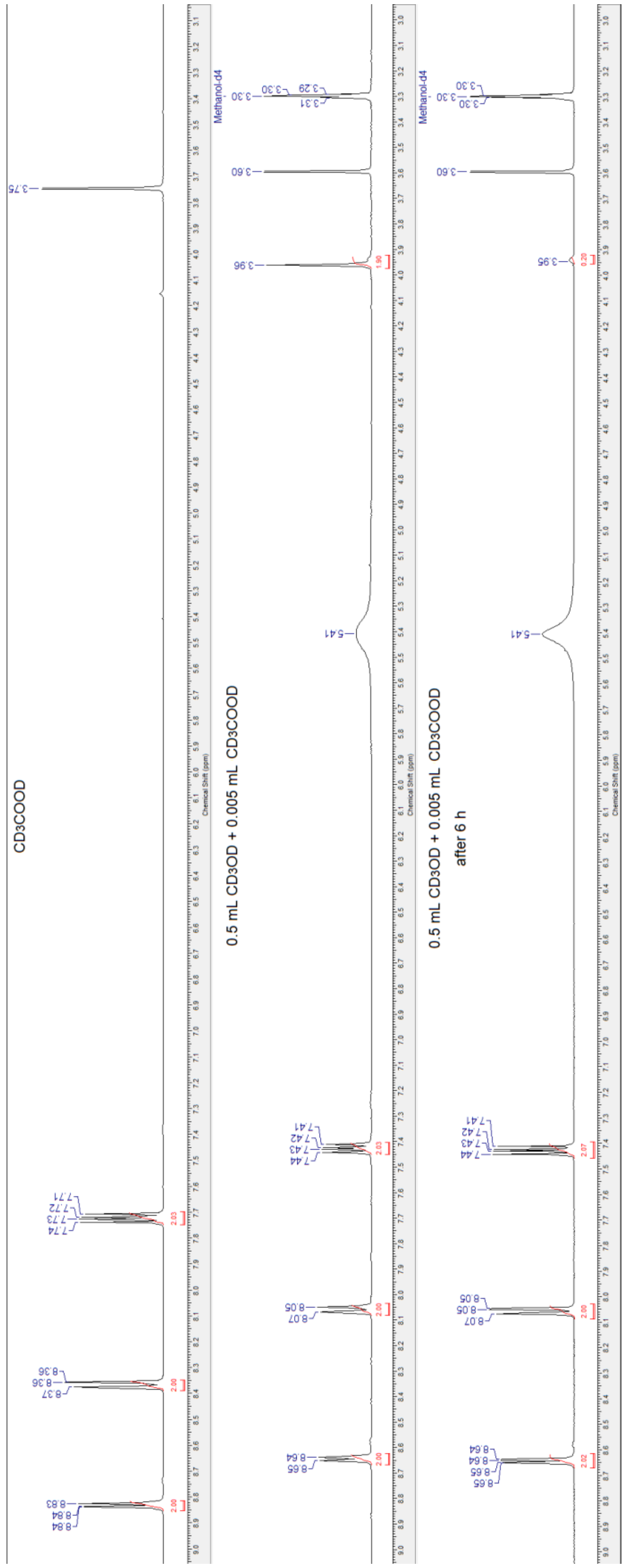

Figure S1: ^{1}H NMR spectra for 4,5-diazafluorene (**1**) in CD_3COOD; in 0.5 mL CD_3OD + 0.005 mL CD_3COOD; in 0.5 mL CD_3OD + 0.005 mL CD_3COOD after 6 h.

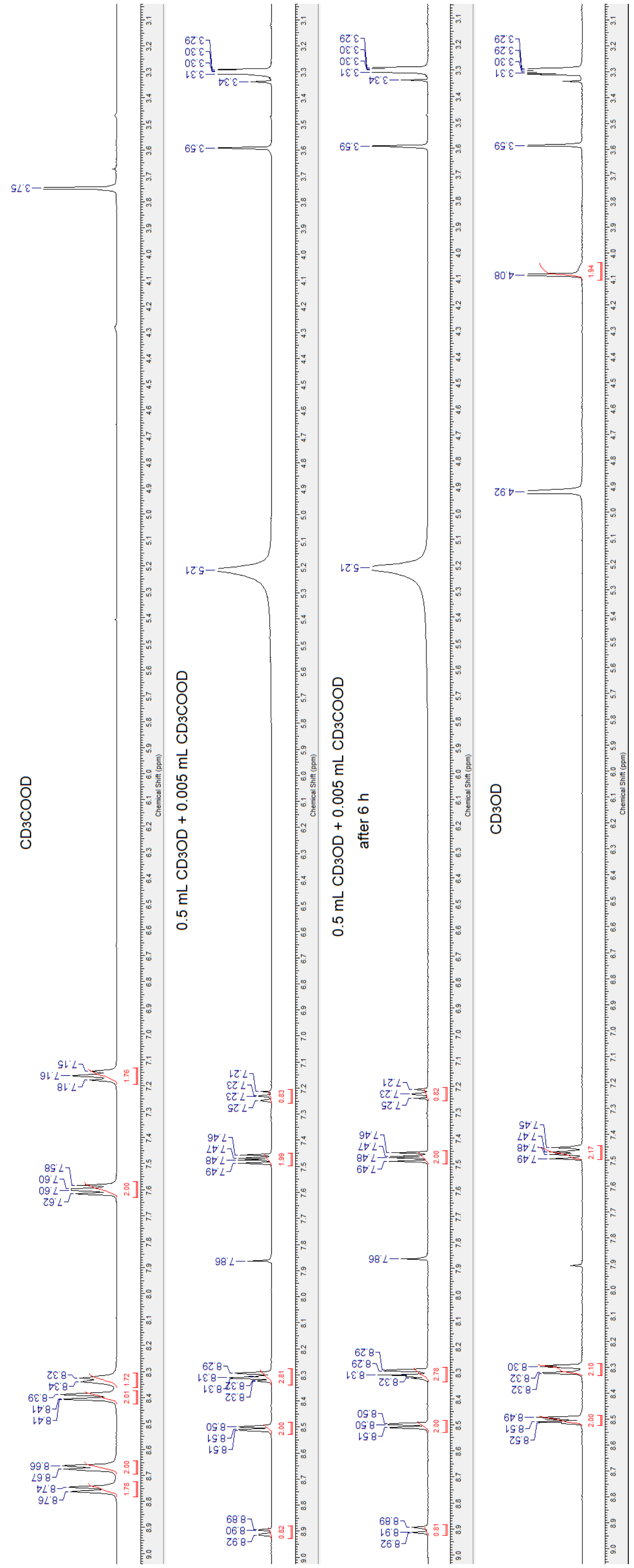

Figure S2: ^{1}H NMR spectra for 1,8-diazafluorene (**2**) in CD_3COOD; in 0.5 mL CD_3OD + 0.005 mL CD_3COOD; in 0.5 mL CD_3OD + 0.005 mL CD_3COOD after 6 h; in CD_3OD.

3. Evaluation of stability of compounds 3b and 4b in acetic acid.

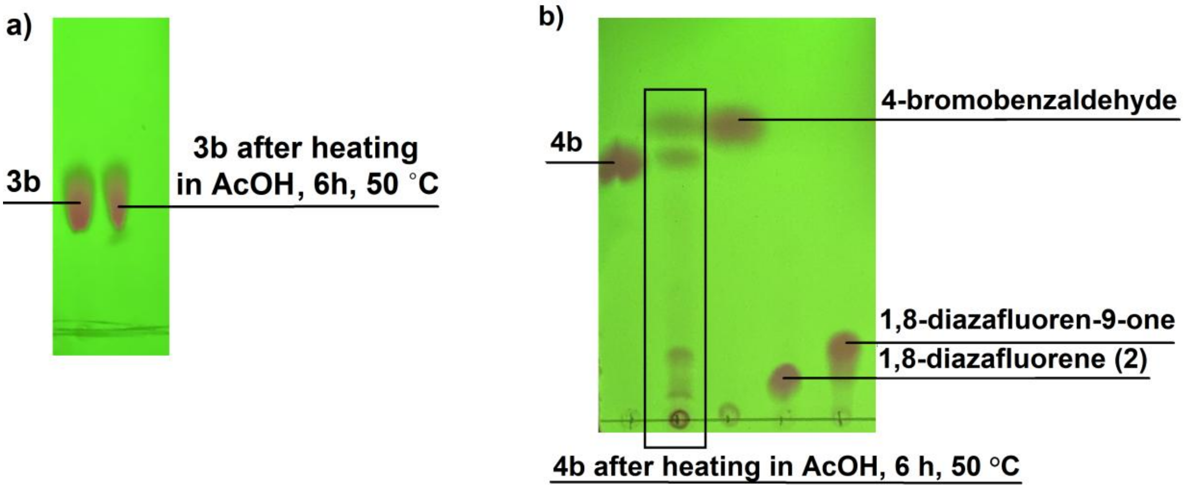

Figure S3: TLC data on compounds **3b** (a) and **4b** (b) after heating at 50 °C in AcOH.

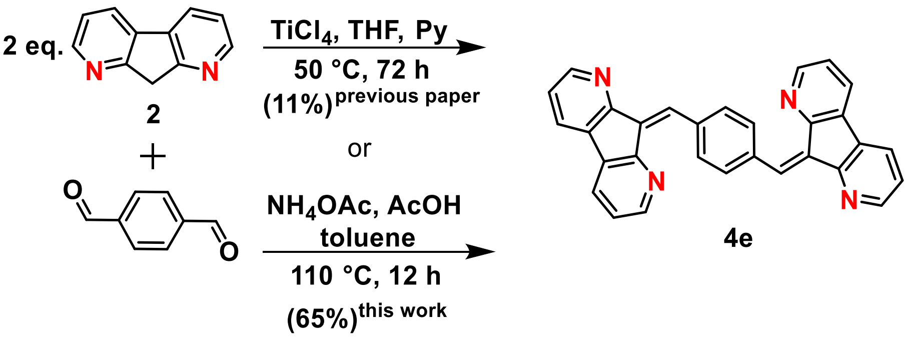

Scheme S1: Condensation approaches of **2** with terephthalaldehyde. Reaction yields are given in parenthesis.

4. X-ray data

Supplementary Note S1: X-ray structure of 1,8-DPDAF

1,8-DPDAF crystallizes as colorless plates. The conformation of the pyridine fragments is non-planar relative to the diazafluorene fragment due to sterical repulsion of pyridine moieties: φ_1 = 68.1(4)° (N_3–C_{13}–C_{12}–C_{10}) and φ_2 = 83.0(4)° (N_4–C_{18}–C_{12}–C_{10}) (Figure S4a). Molecules are packed into stacks along the a-axis due to π-stacking interactions between diazafluorenes and hydrogen bonds with water molecule (Figure S4b). The structure also has lattice channels of approximately 984 Å^3 with removed disordered water molecules (Figure S4c).

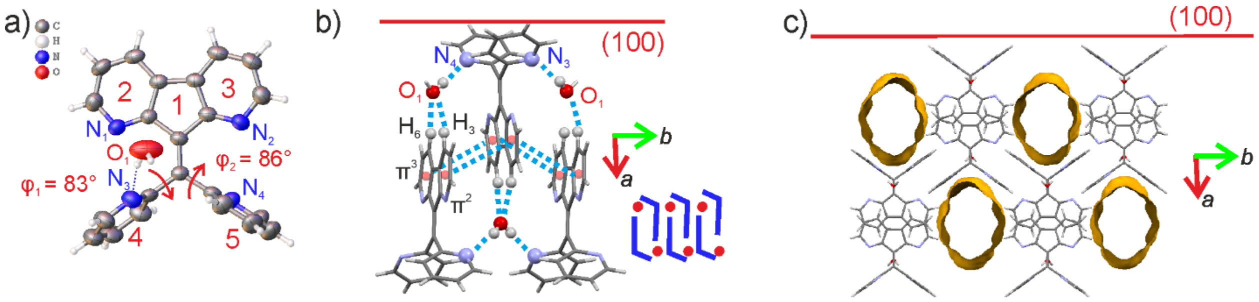

Figure S4: Molecular structure, atom and cycle numbering of 1,8-DPDAF with anisotropic displacement ellipsoids drawn at a 50% probability level (a); crystal structure fragment with π-stacking of 1,8-DPDAF (b). Lattice channels of approximately 405 Å^3 as mapped by the contact yellow surface of a probe with a radius of 1.2 Å. Dashed blue lines represent noncovalent interactions. The arrows indicate the orientation of crystallographic axes. Red line shows the (001) plane. The schematic packing motif is shown in blue, water molecules are shown as red dots.

Table S1: Crystal data, data collection, and structure refinement parameters for 4,5-DPDAF, Zn–4,5-DPDAF and 1,8- DPDAF.

	**4,5-DPDAF**	**Zn–4,5-DPDAF**	**1,8-DPDAF**
Chemical formula	$C_{22}H_{14}N_4$	$C_{44}H_{28}Cl_2N_8Zn_2$·2($ZnCl_3$ H_2O)·1.5(O)	$C_{22}H_{14}N_4$·H_2O·1[H_2O]
M_r	334.37	1273.85	370.40
Crystal system, space group	monoclinic, *C*2/*c*	orthorhombic, *Pbam*	orthorhombic, *Pbca*
a, *b*, *c* (Å)	13.7111 (8),<br>10.5932 (6),<br>23.4823 (15)	15.659 (6),<br>12.596 (4),<br>13.175 (5)	19.9962 (14),<br>7.1401 (7),<br>29.647 (2)
β (°)	105.598 (7)	90	90
V (Å^3)	3285.1 (4)	2598.7 (16)	4232.9 (6)
Z/Z'	8/1	2/0.5	2/1
D_{calcd} (g cm^{-3})	1.352	1.628	1.162
Solvent mask in 1 void per unit cell			984 A^3 (136 e)
No. of measured, independent and observed [$I > 2\sigma(I)$] reflections	16194, 2971, 2441	7194, 2290, 1449	49107, 3856, 2659
R_{int}	0.031	0.081	0.102
$R[F^2 > 2\sigma(F^2)]$, $wR(F^2)$, S	0.037, 0.097, 1.03	0.099, 0.288, 1.13	0.075, 0.204, 1.05
$\Delta\rho_{max}$, $\Delta\rho_{min}$ (e Å^{-3})	0.12, −0.18	2.28, −1.28	0.23, −0.20

Table S2: Noncovalent interactions for 4,5-DPDAF, Zn–4,5-DPDAF and 1,8-DPDAF.

	Interactions	Cg–Cg, Å	D_{pln}, Å	π–π, °
**4,5-DPDAF**	$\pi^1\cdots\pi^2$	3.5029(9)	3.4840(6)	1.17(7)
	$\pi^1\cdots\pi^3$	3.5424(9)	3.4135(6)	1.15(7)
	$\pi^2\cdots\pi^2$	3.9452(9)	3.4765(6)	0.00(7)
	$\pi^3\cdots\pi^3$	3.6234(9)	3.3987(6)	0.00(7)
	$\pi^4\cdots\pi^4$	3.7687(9)	3.5831(6)	17.40(7)
		H···Cg (Å)	D_{pln} (Å)	C–H···Cg (°)
	C_{16}–H···π^5	3.09	3.08	134
	C_3–H···π^4 (intra)	2.82	2.55	145
	C_7-H···π^5 (intra)	2.92	2.60	146
		H···A (Å)	D···A (Å)	D–H···A (°)
	C_{14}–H···N_1	2.52	3.393(2)	157
	C_{19}–H···N_2	2.72	3.475(2)	139
**Zn-4,5-DPDAF**		Cg–Cg, Å	D_{pln}, Å	π–π, °
	$\pi^1\cdots\pi^1$	3.447(6)	3.411(5)	0.0(6)
	$\pi^1\cdots\pi^2$	3.949(6)	3.406(5)	2.3(5)
	$\pi^2\cdots\pi^2$	3.553(6)	3.41	3.414(4)
	$\pi^2\cdots\pi^3$	4.37	4.08	39
		H/Cl···Cg (Å)	D_{pln} (Å)	C–H/Cl···Cg (°)
	C_1–H···π^2	3.09	2.99	109
	C_9–H···π^3 (intra)	2.98	2.53	139
	Zn_1–Cl_1···π^1	3.737(7)	3.291	100.64(18)
		Cl···O (Å)	Zn–Cl···O (°)	Cl···O–Zn (°)
	Cl_2···O_1	3.20	113	125
**1,8-DPDAF**		Cg–Cg, Å	D_{pln}, Å	π–π, °
	$\pi^2\cdots\pi^2$	4.0132(15)	3.4140(10)	6.19(12)
	$\pi^3\cdots\pi^3$	4.0391(15)	3.3892(10)	7.14(12)
		H···A (Å)	D···A (Å)	D–H···A (°)
	O_1–H_{1a}···N_4	2.09	2.941(4)	179
	O_1–H_{1b}···N_3	2.16	2.970(4)	160
	C_3–H···O_1	2.58	3.476(4)	162
	C_6–H···O_1	2.62	3.512(4)	161

Cg is the aromatic ring center; D_{pln} is the nearest distance between H-atom and aromatic ring plane. Atom labels are corresponding to the label scheme in Figure 2 and Figure S4.

5. Spectroscopy data

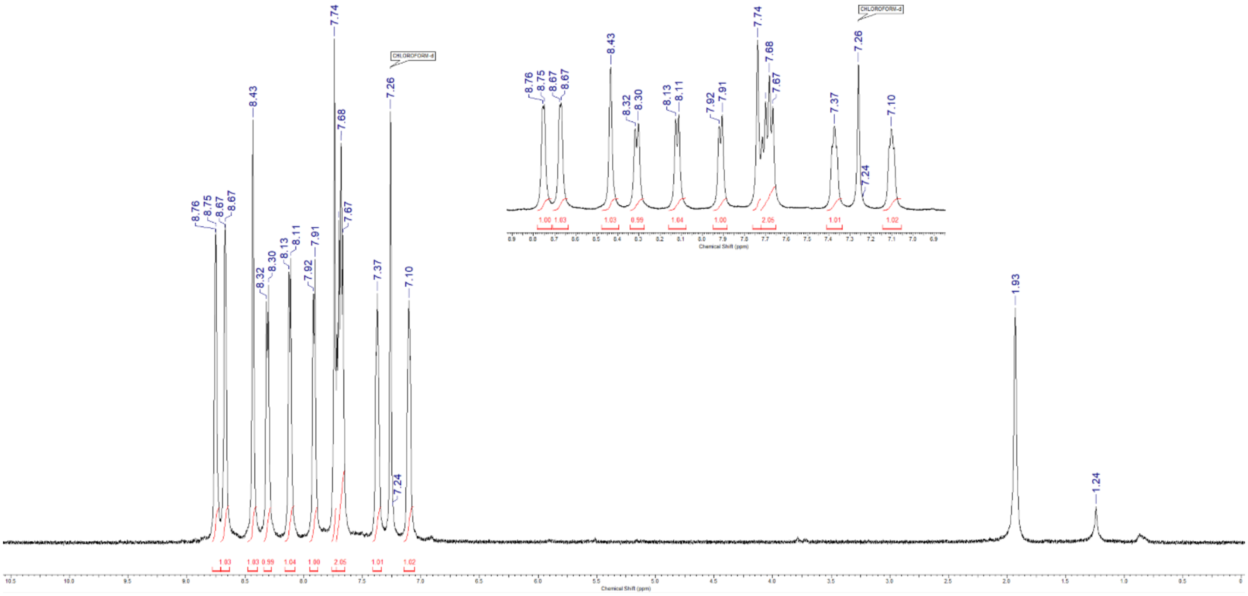

Figure S5: ^{1}H NMR spectrum of 9-(3-nitrobenzylidene)-9*H*-4,5-diazafluorene (**3a**) in $CDCl_3$.

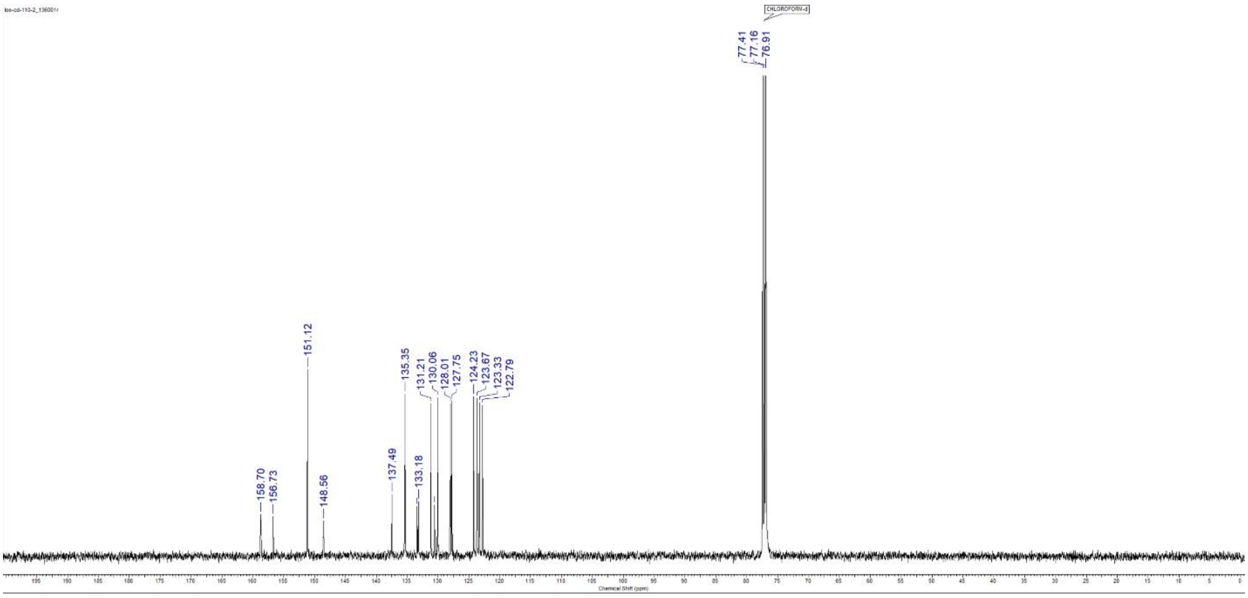

Figure S6: ^{13}C NMR spectrum of 9-(3-nitrobenzylidene)-9*H*-4,5-diazafluorene (**3a**) in $CDCl_3$.

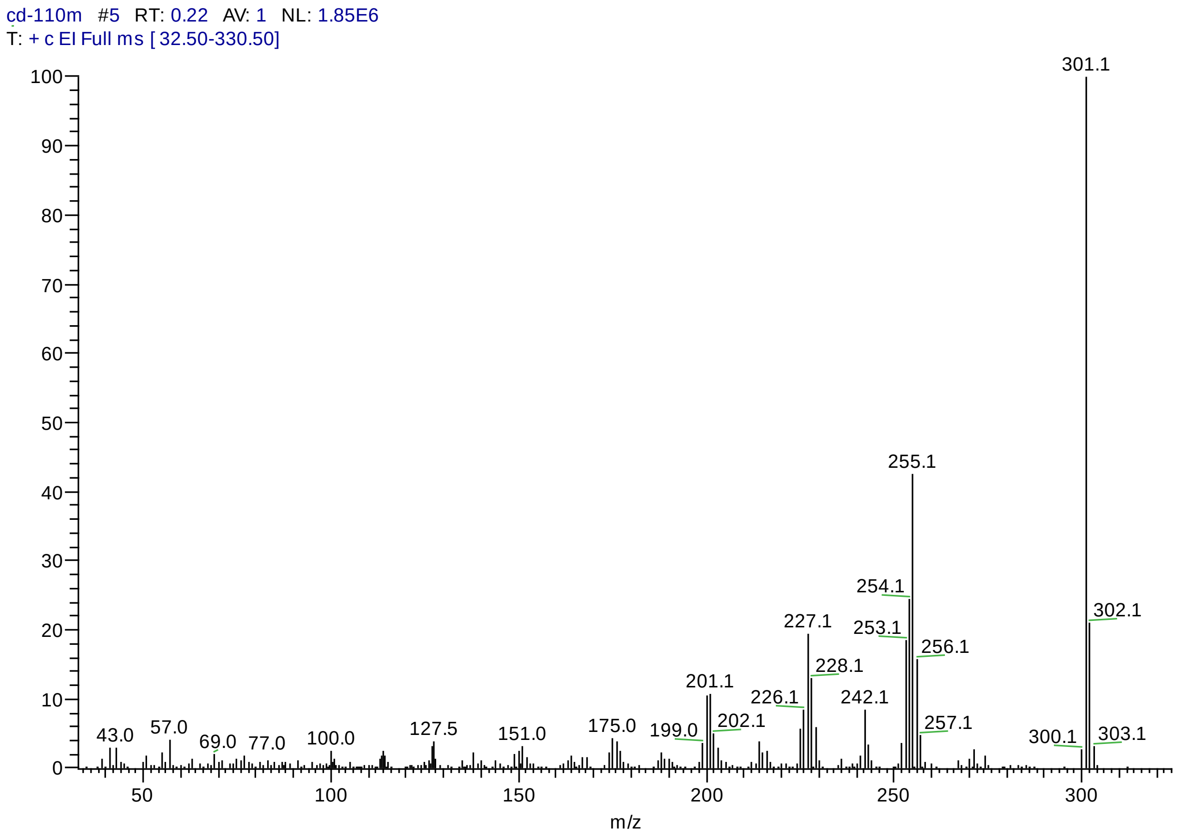

Figure S7: HRMS overview spectrum of 9-(3-nitrobenzylidene)-9*H*-4,5-diazafluorene (**3a**) (T_{source} = 70 °C, T_{probe} = 200 °C).

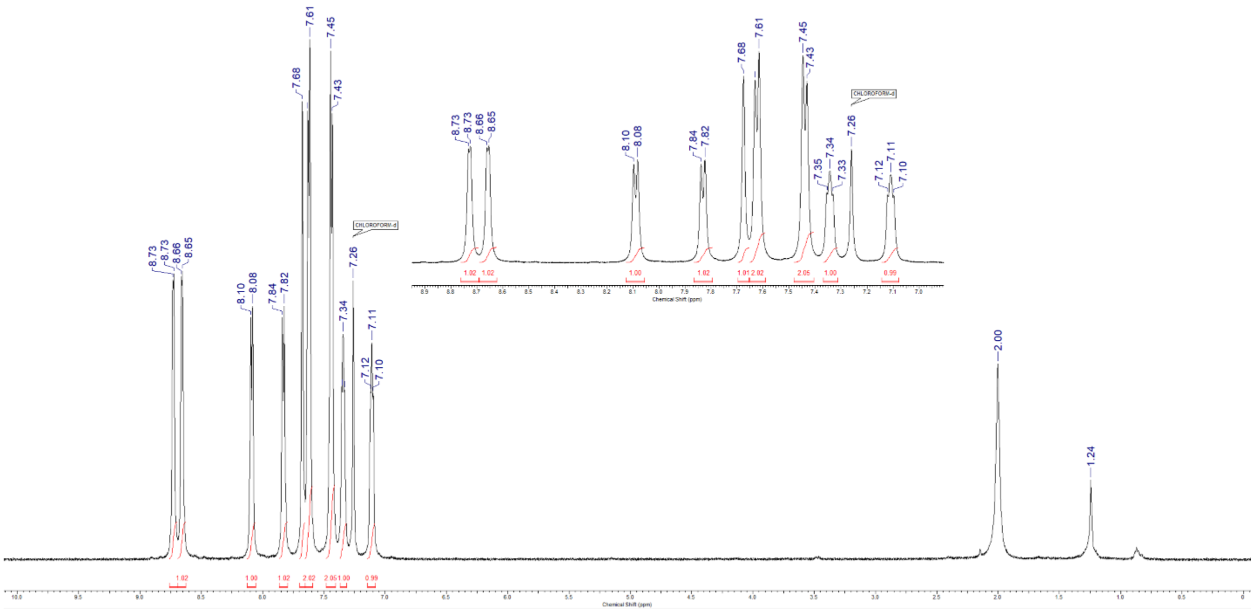

Figure S8: ^{1}H NMR spectrum of 9-(4-bromobenzylidene)-9*H*-4,5-diazafluorene (**3b**) in $CDCl_3$.

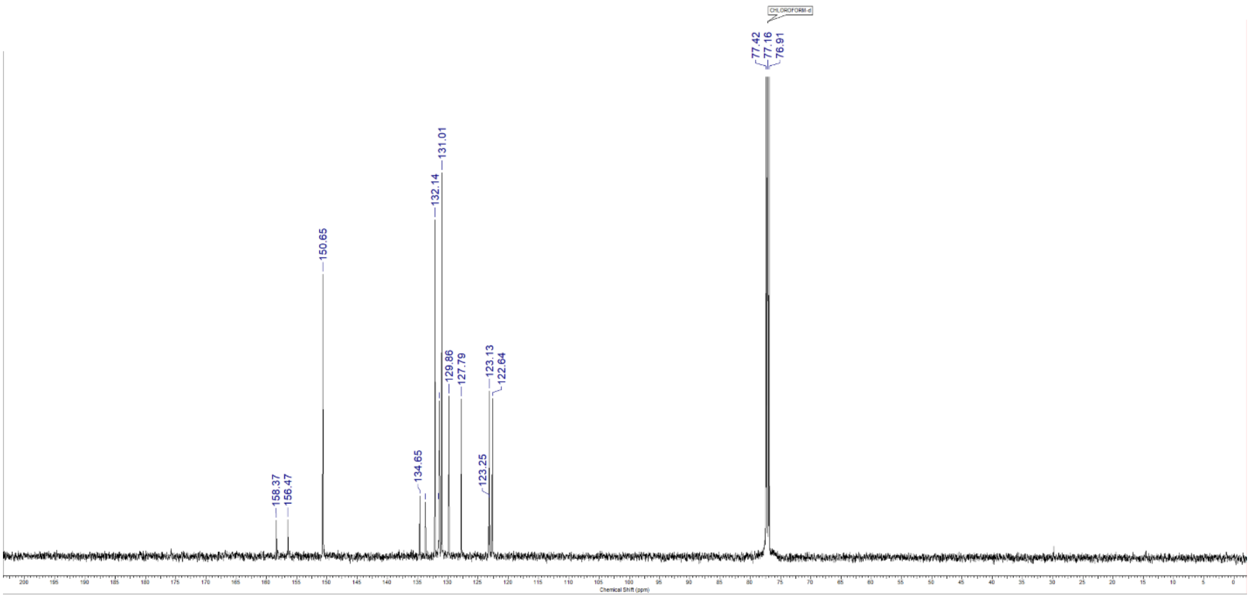

Figure S9: ^{13}C NMR spectrum of 9-(4-bromobenzylidene)-9*H*-4,5-diazafluorene (**3b**) in $CDCl_3$.

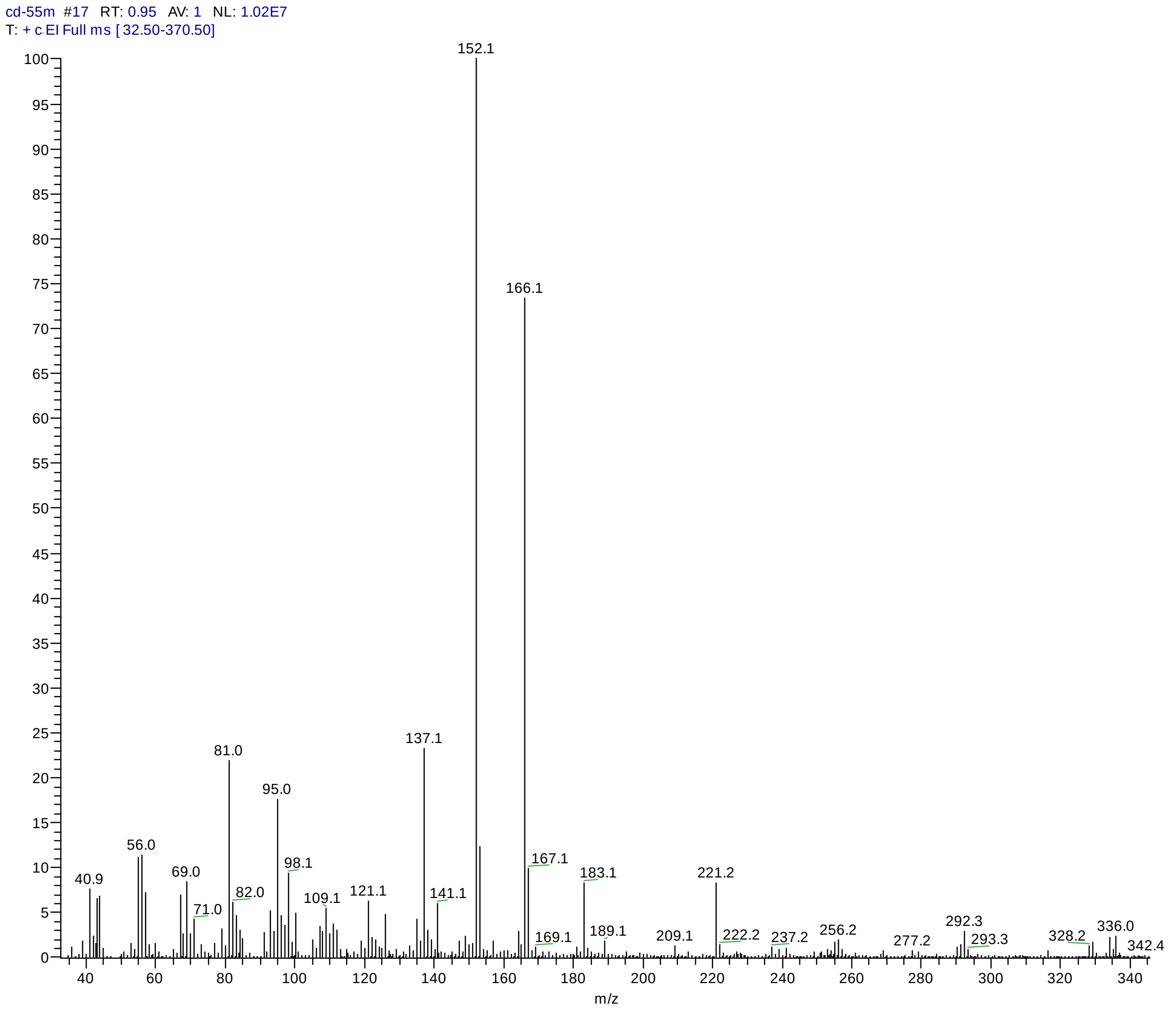

Figure S10: HRMS overview spectrum of 9-(4-bromobenzylidene)-9*H*-4,5-diazafluorene (**3b**) (T_{source} = 75 °C, T_{probe} = 160 °C).

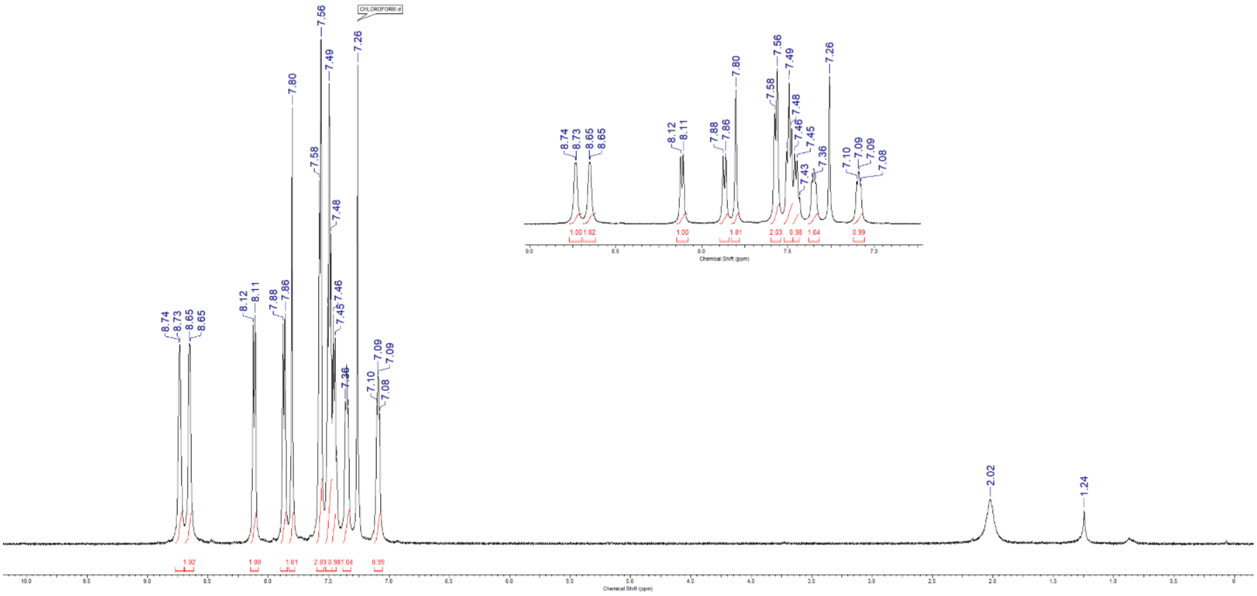

Figure S11: ^{1}H NMR spectrum of 9-benzylidene-9*H*-4,5-diazafluorene (**3c**) in $CDCl_3$.

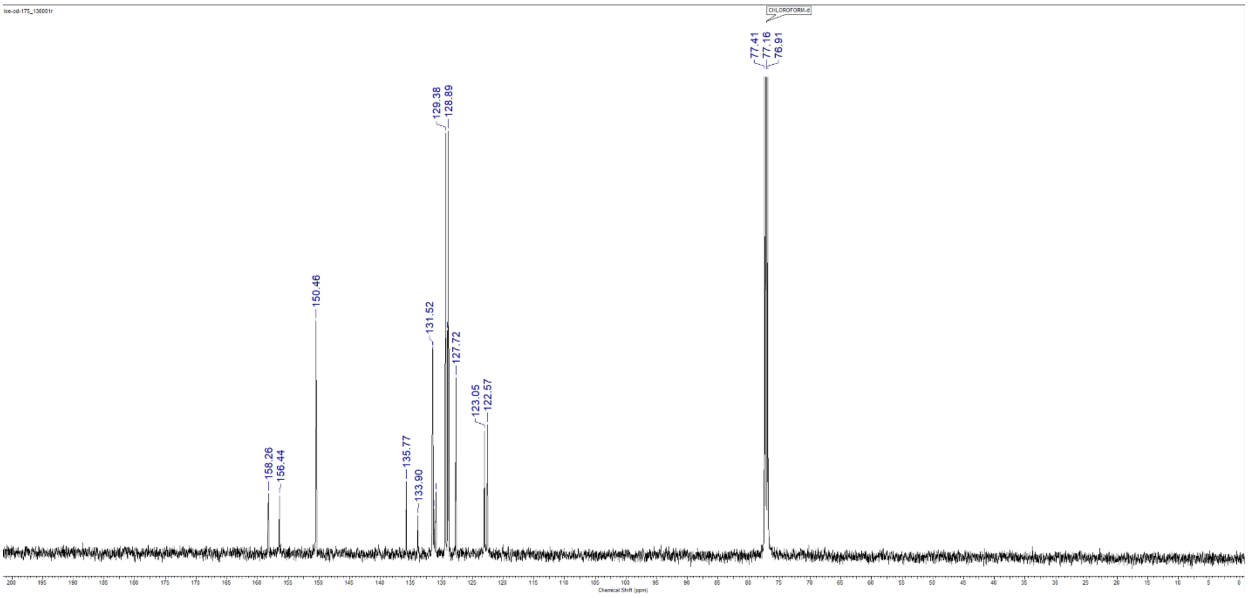

Figure S12: ^{13}C NMR spectrum of 9-benzylidene-9*H*-4,5-diazafluorene (**3c**) in $CDCl_3$.

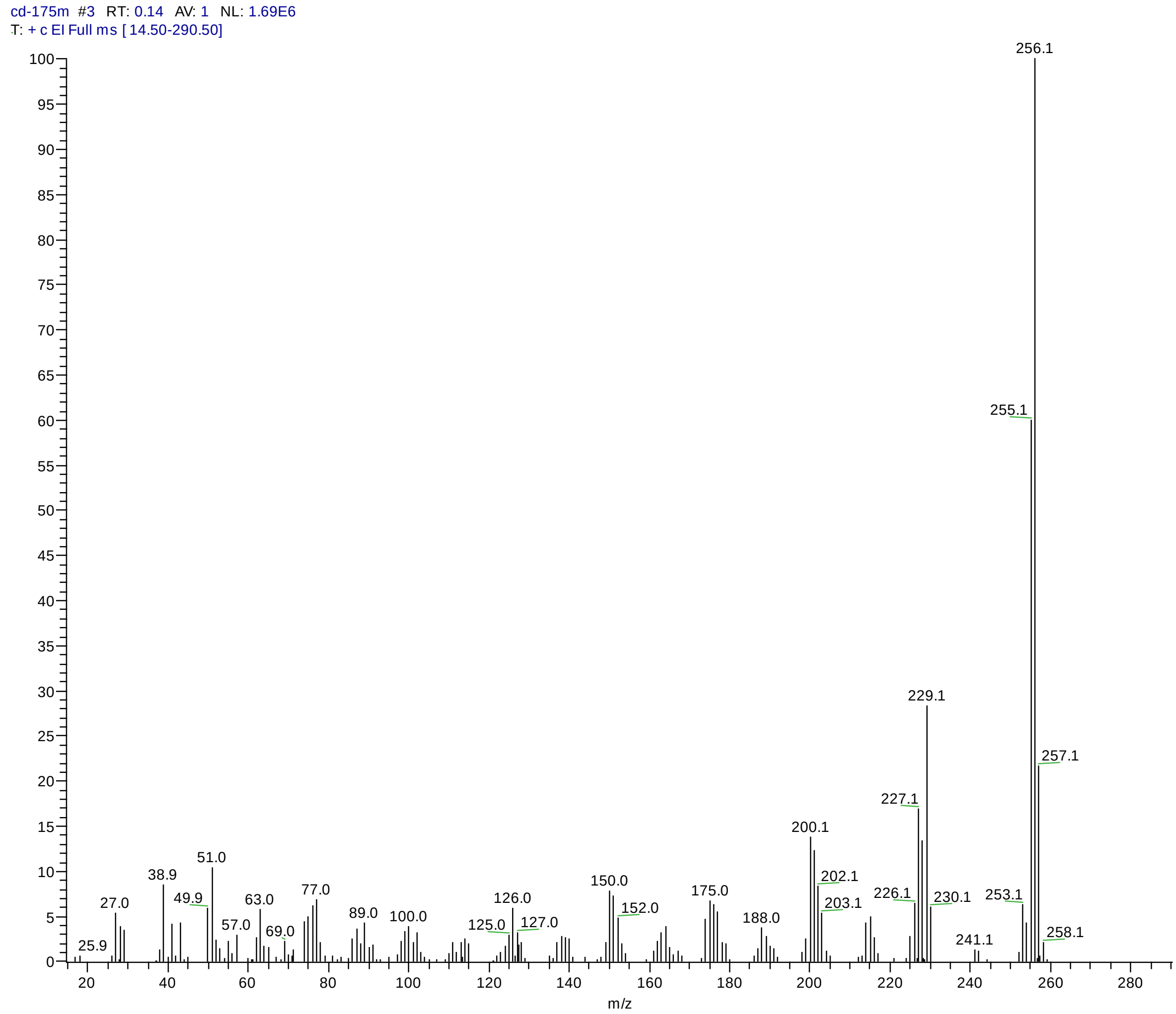

Figure S13: HRMS overview spectrum of 9-benzylidene-9*H*-4,5-diazafluorene (**3c**) (T_{source} = 50 °C, T_{probe} = 220 °C).

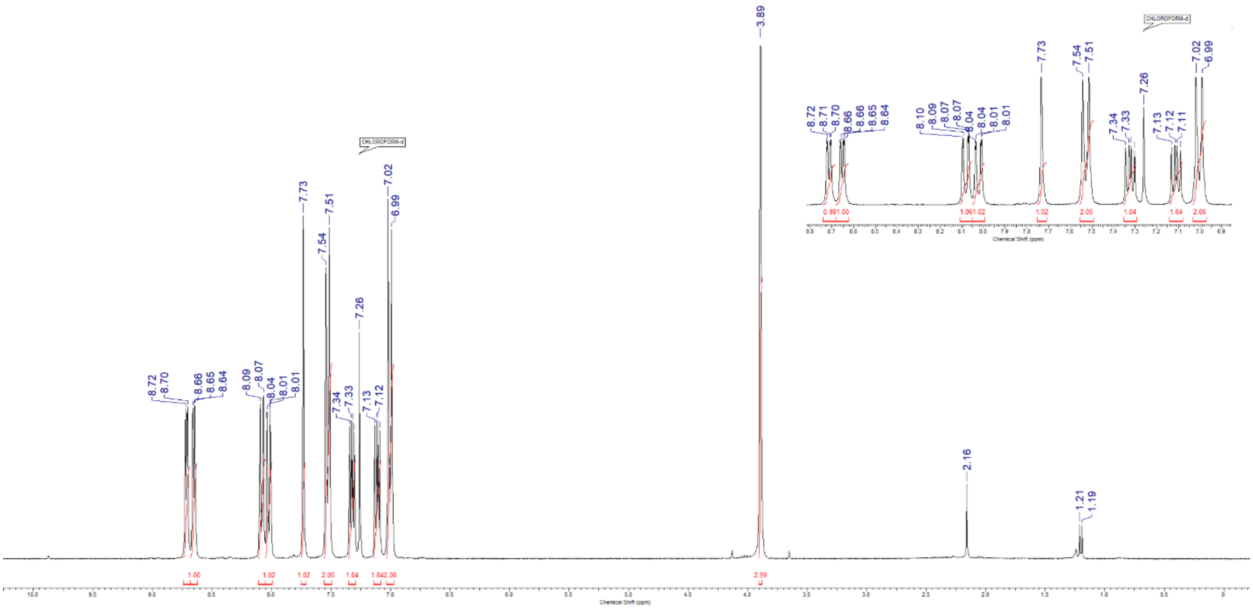

Figure S14: ^{1}H NMR spectrum of 9-(4-methoxybenzylidene)-9*H*-4,5-diazafluorene (**3d**) in $CDCl_3$.

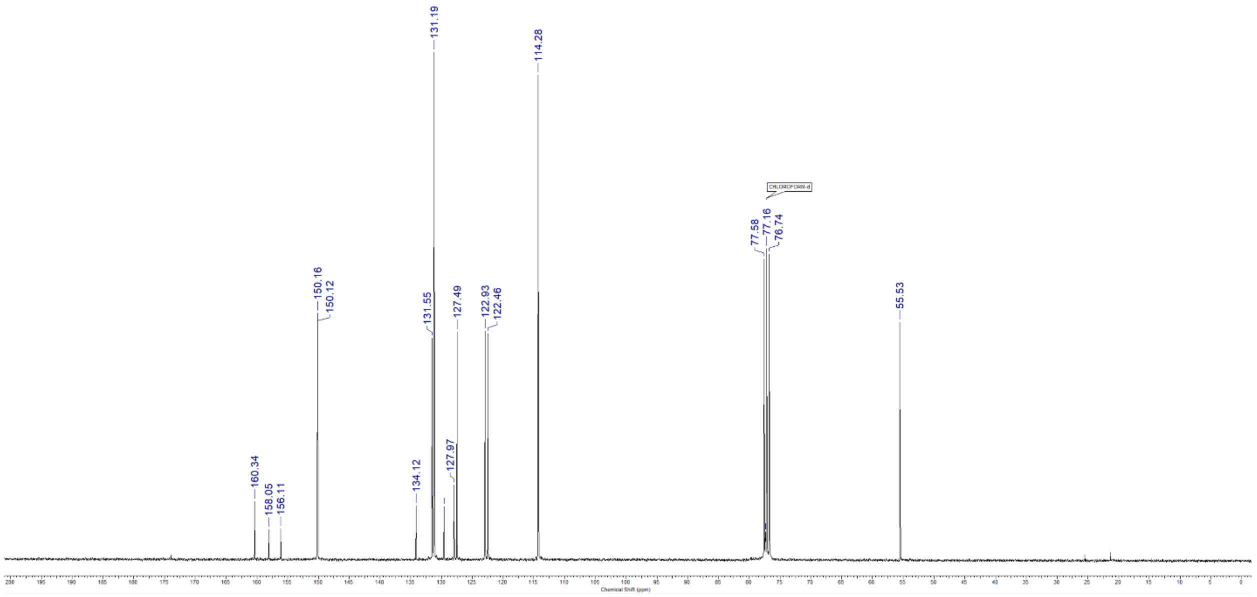

Figure S15: ^{13}C NMR spectrum of 9-(4-methoxybenzylidene)-9*H*-4,5-diazafluorene (**3d**) in $CDCl_3$.

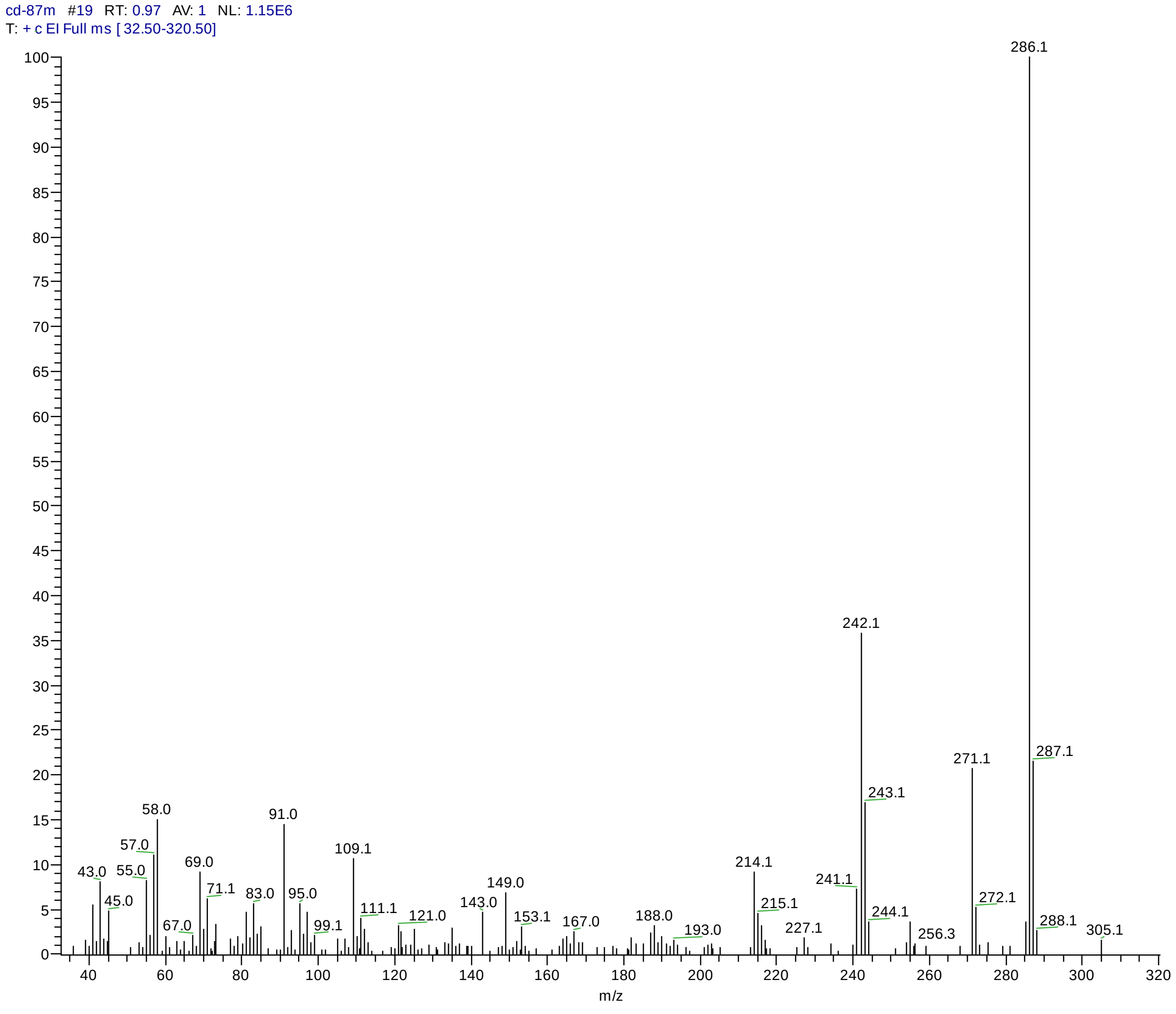

Figure S16: HRMS overview spectrum of 9-(4-methoxybenzylidene)-9*H*-4,5-diazafluorene (**3d**) (T_{source} = 95 °C, T_{probe} = 200 °C).

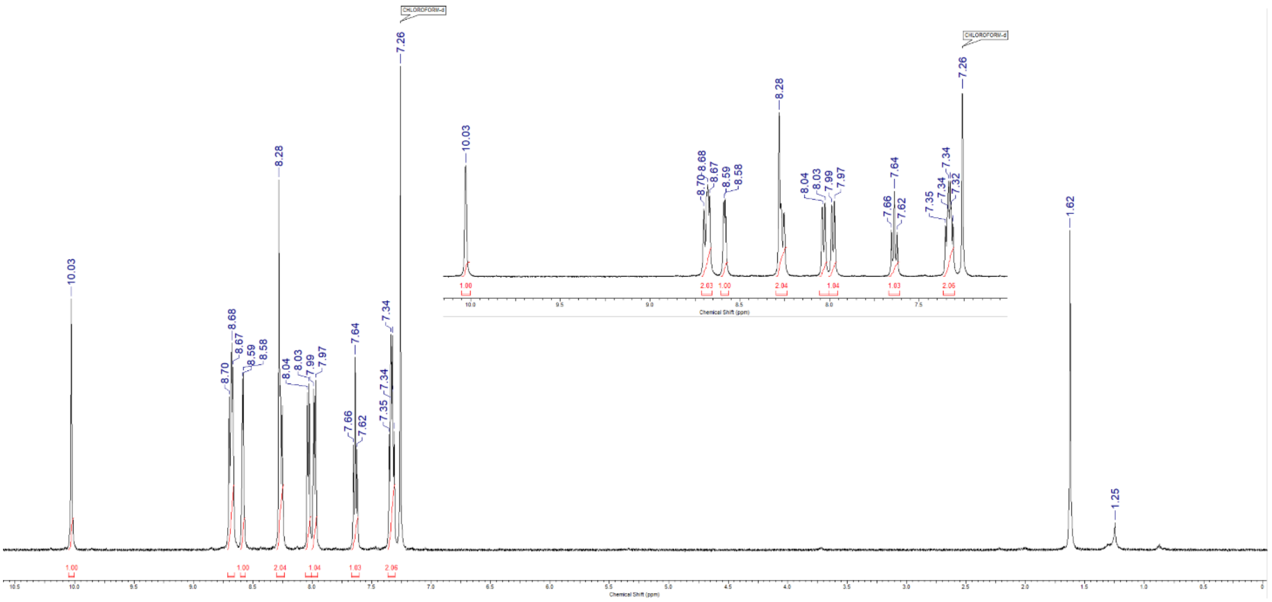

Figure S17: ^{1}H NMR spectrum of 9-(3-nitrobenzylidene)-9*H*-1,8-diazafluorene (**4a**) in $CDCl_3$.

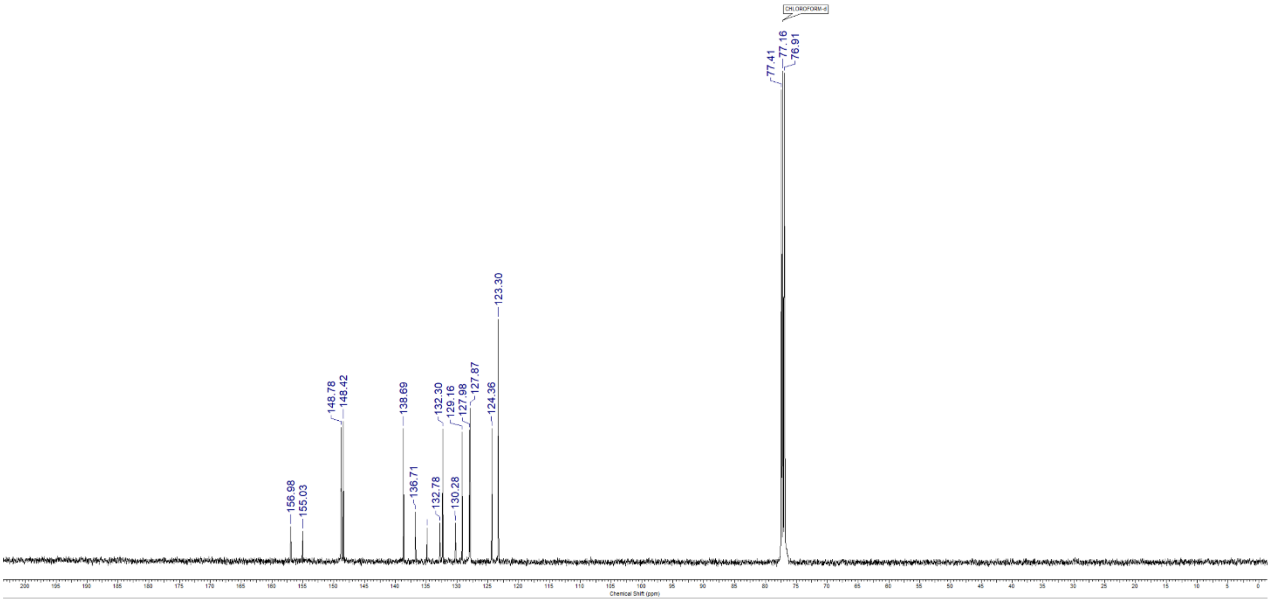

Figure S18: ^{13}C NMR spectrum of 9-(3-nitrobenzylidene)-9*H*-1,8-diazafluorene (**4a**) in $CDCl_3$.

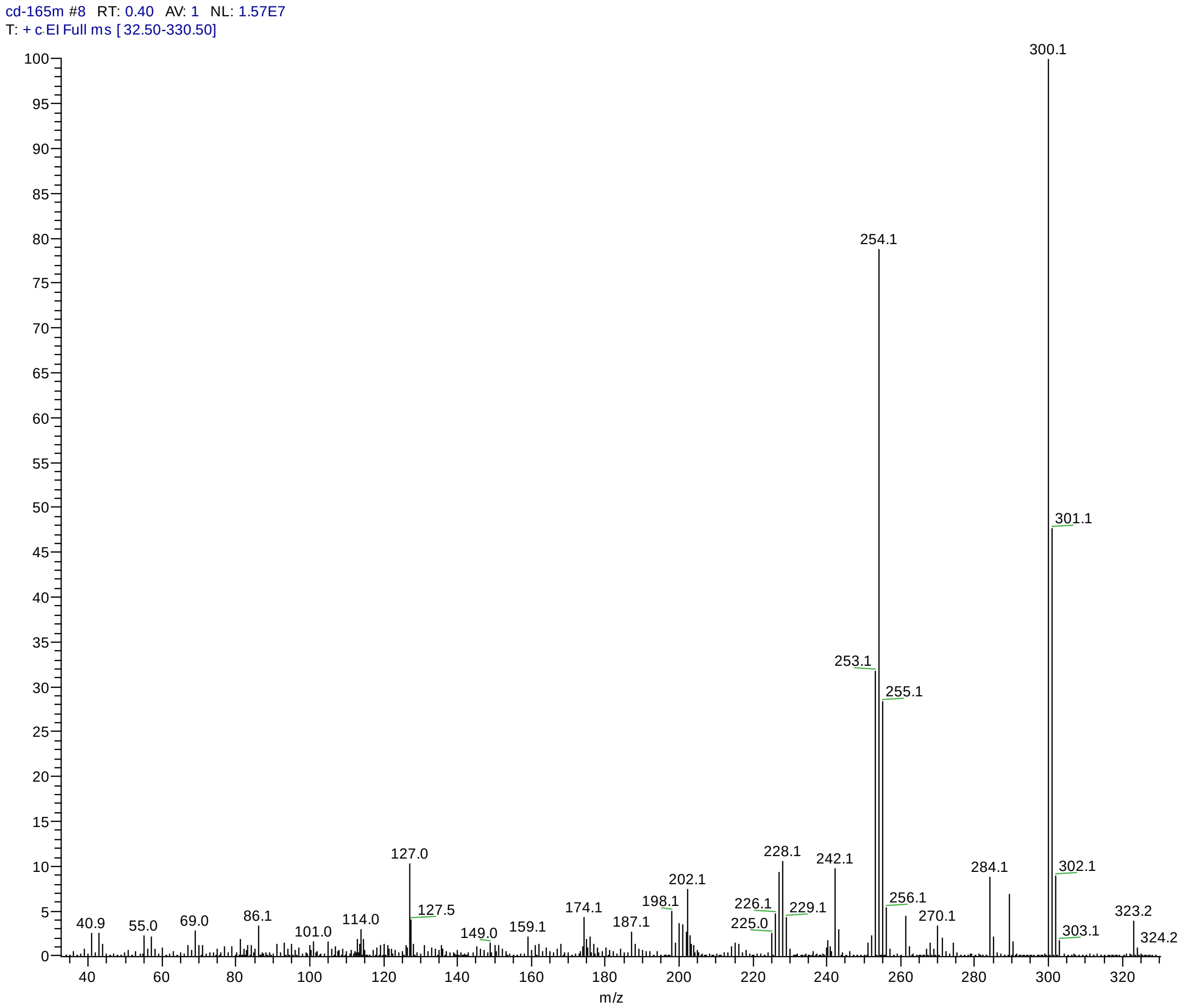

Figure S19: HRMS overview spectrum of 9-(3-nitrobenzylidene)-9*H*-1,8-diazafluorene (**4a**) (T_{source} = 70 °C).

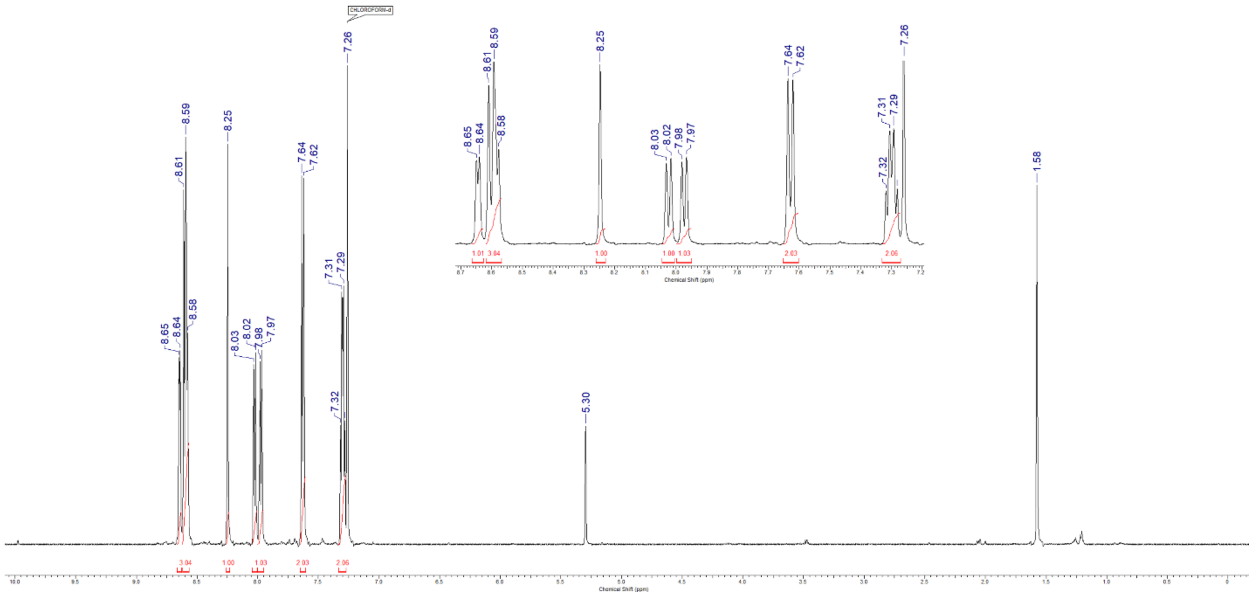

Figure S20: ^{1}H NMR spectrum of 9-(4-bromobenzylidene)-9*H*-1,8-diazafluorene (**4b**) in $CDCl_3$.

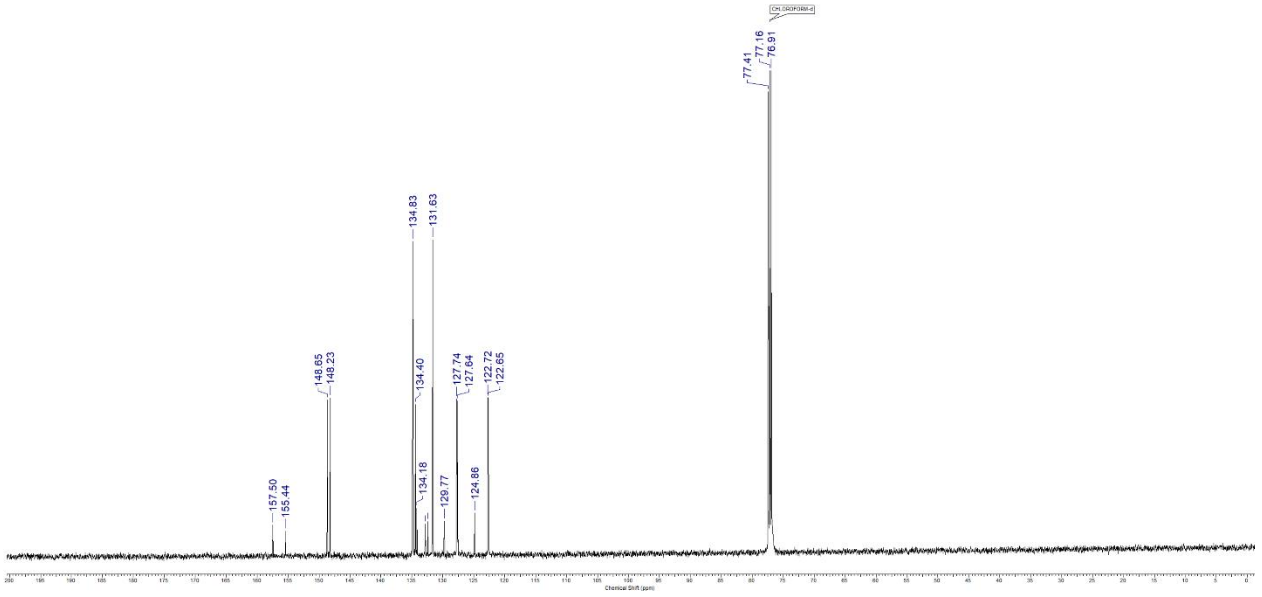

Figure S21: ^{13}C NMR spectrum of 9-(4-bromobenzylidene)-9*H*-1,8-diazafluorene (**4b**) in $CDCl_3$.

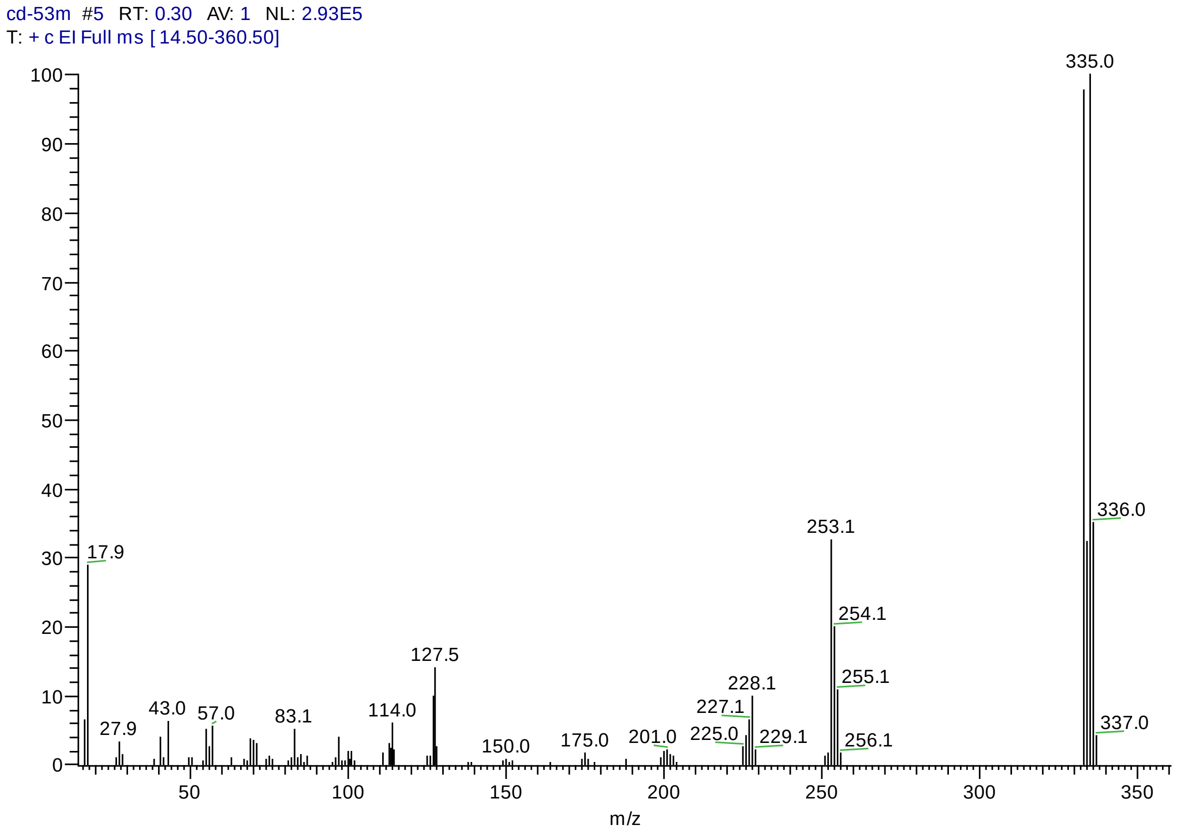

Figure S22: HRMS overview spectrum of 9-(4-bromobenzylidene)-9*H*-1,8-diazafluorene (**4b**) (T_{source} = 105 °C).

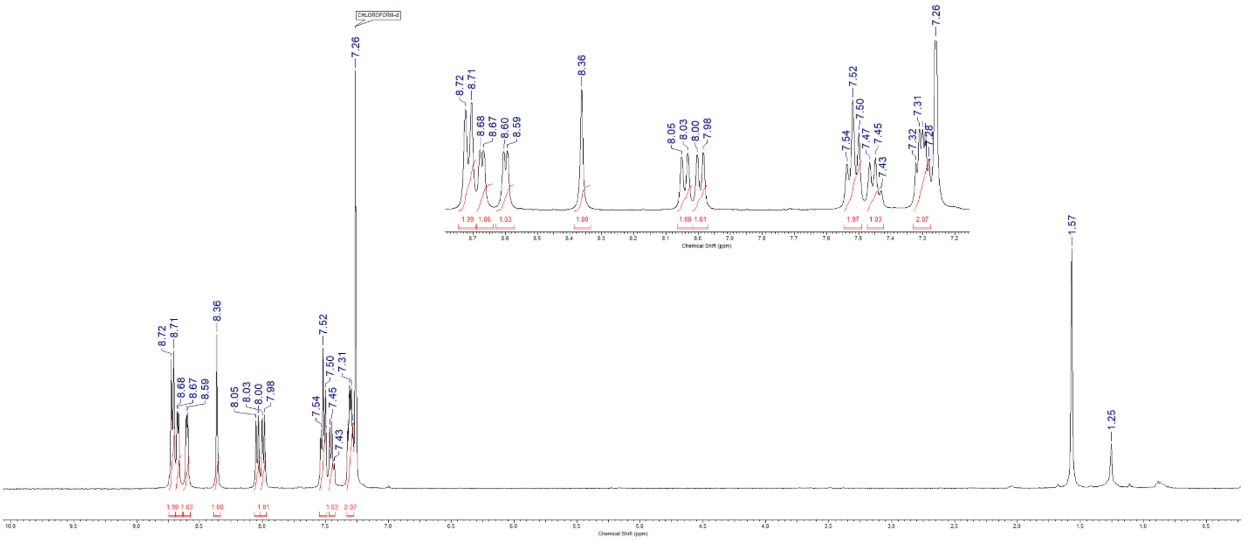

Figure S23: ^{1}H NMR spectrum of 9-benzylidene-9*H*-1,8-diazafluorene (**4c**) in $CDCl_3$.

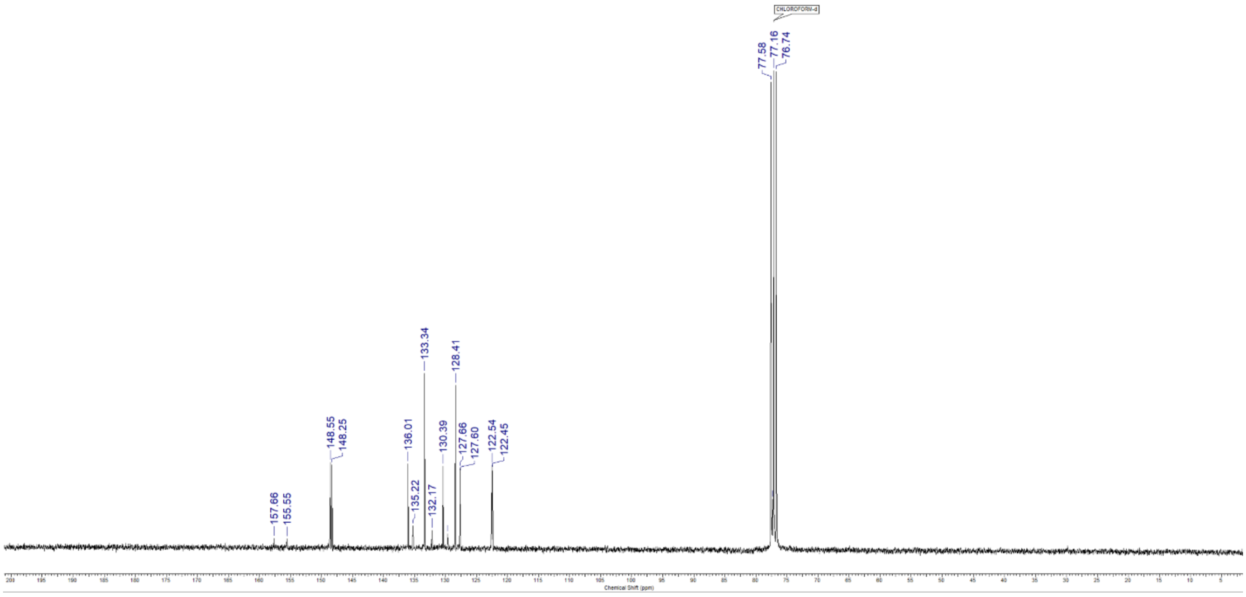

Figure S24: ^{13}C NMR spectrum of 9-benzylidene-9*H*-1,8-diazafluorene (**4c**) in $CDCl_3$.

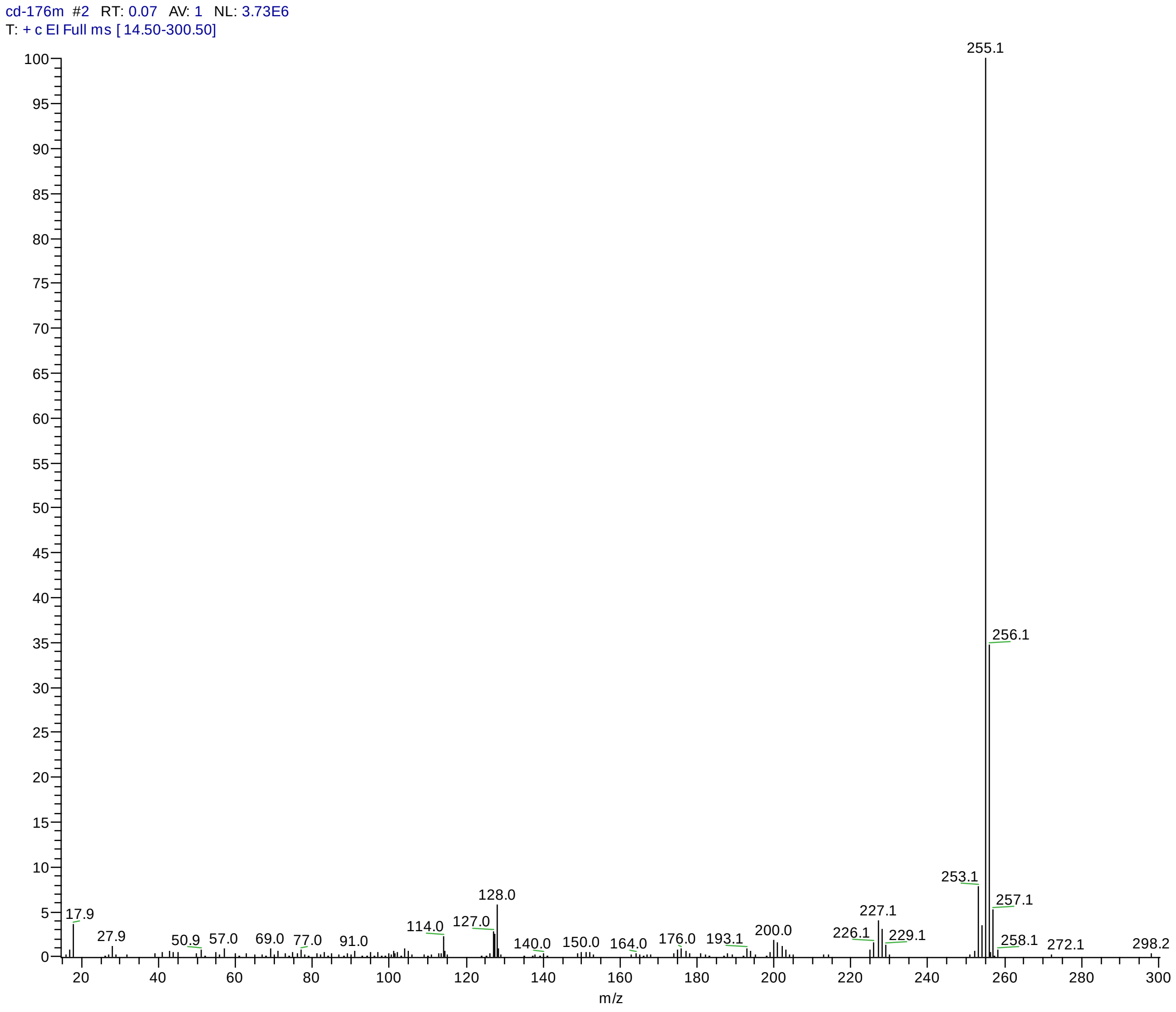

Figure S25: HRMS overview spectrum of 9-benzylidene-9*H*-1,8-diazafluorene (**4c**) (T_{source} = 50 °C, T_{probe} = 200 °C).

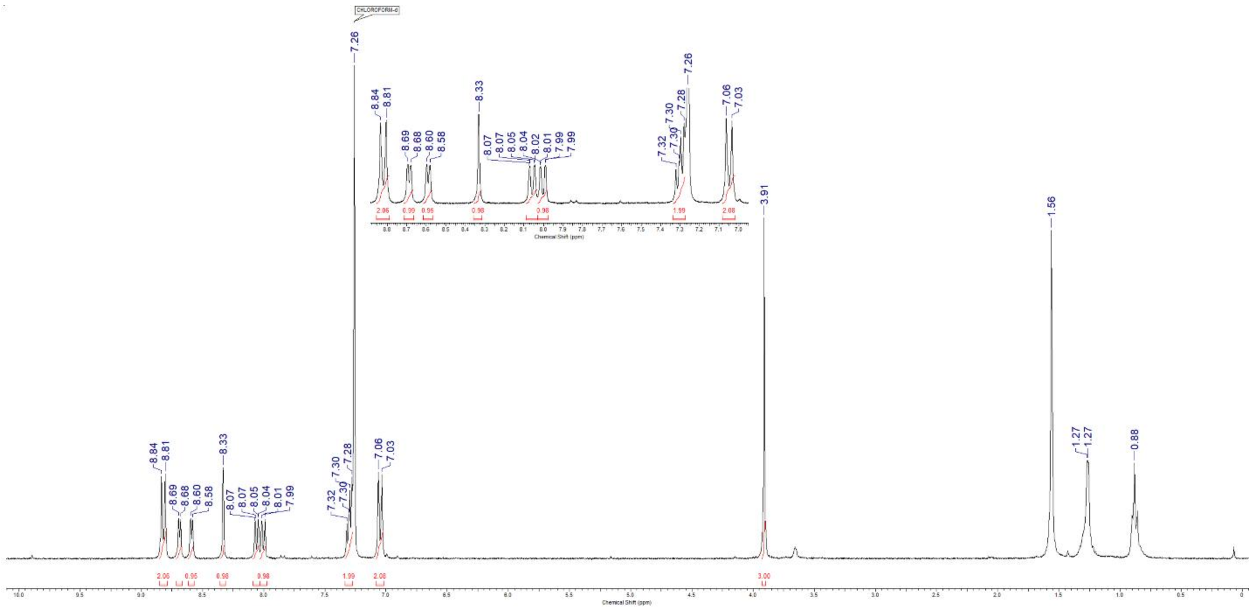

Figure S26: ^{1}H NMR spectrum of 9-(4-methoxybenzylidene)-9*H*-1,8-diazafluorene (**4d**) in $CDCl_3$.

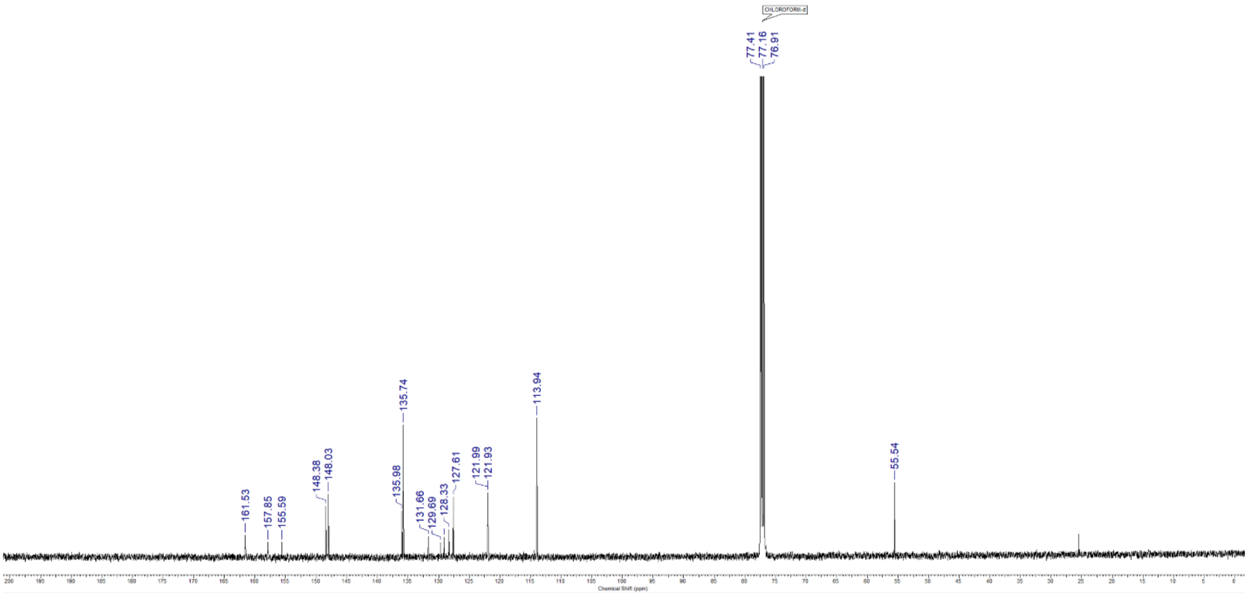

Figure S27: ^{13}C NMR spectrum of 9-(4-methoxybenzylidene)-9*H*-1,8-diazafluorene (**4d**) in $CDCl_3$.

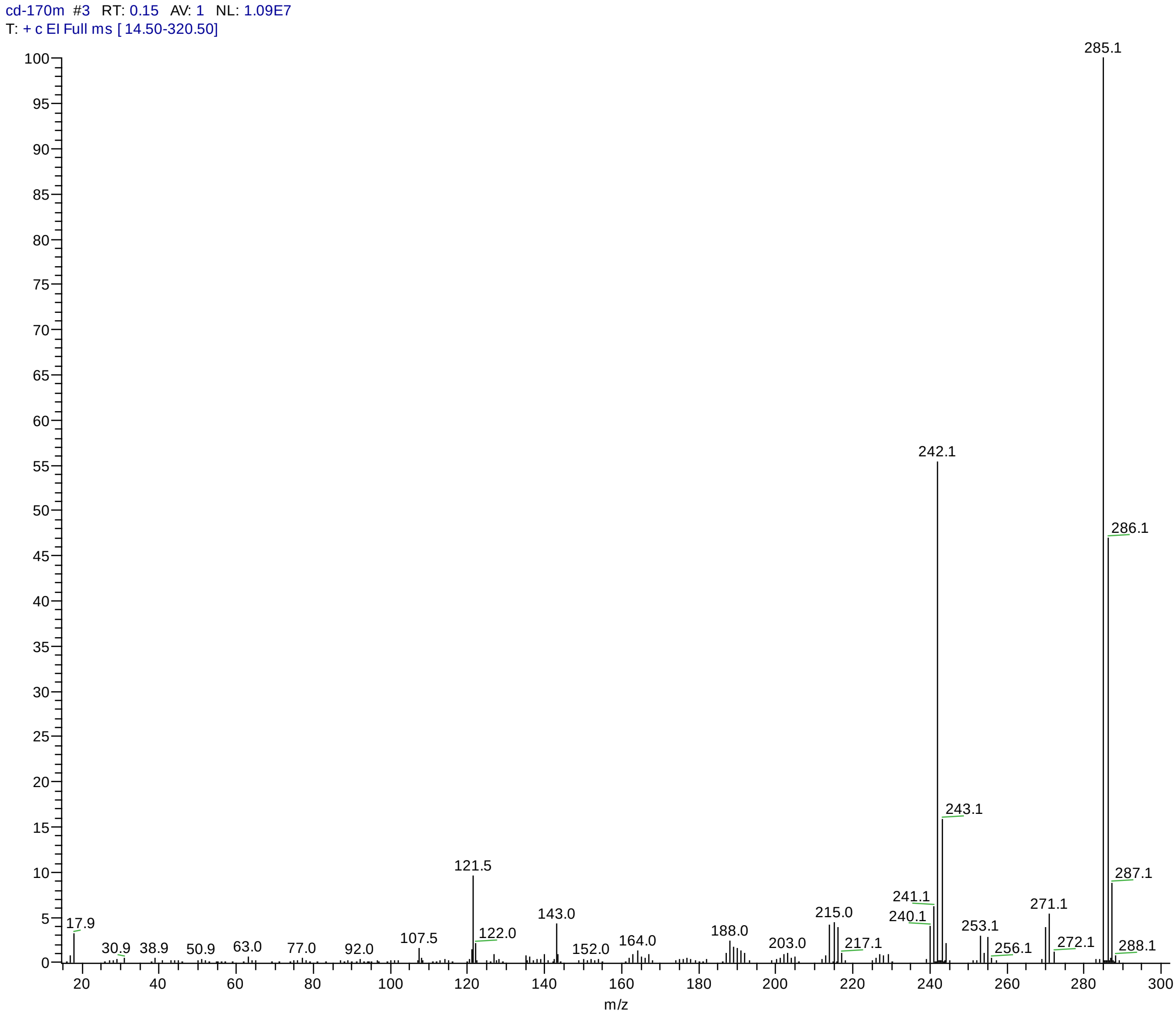

Figure S28: HRMS overview spectrum of 9-(4-methoxybenzylidene)-9*H*-1,8-diazafluorene (**4d**) (T_{source} = 60 °C, T_{probe} = 200 °C).

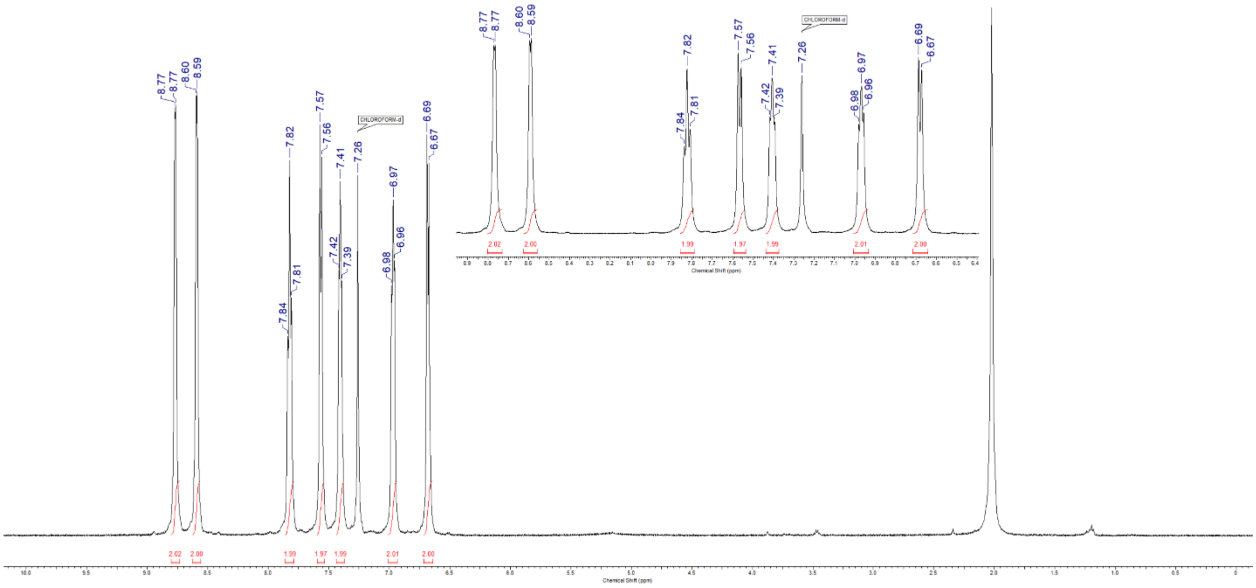

Figure S29: ^{1}H NMR spectrum of 9-(di(pyridin-2-yl)methylene)-9*H*-4,5-diazafluorene (4,5-DPDAF) in $CDCl_3$.

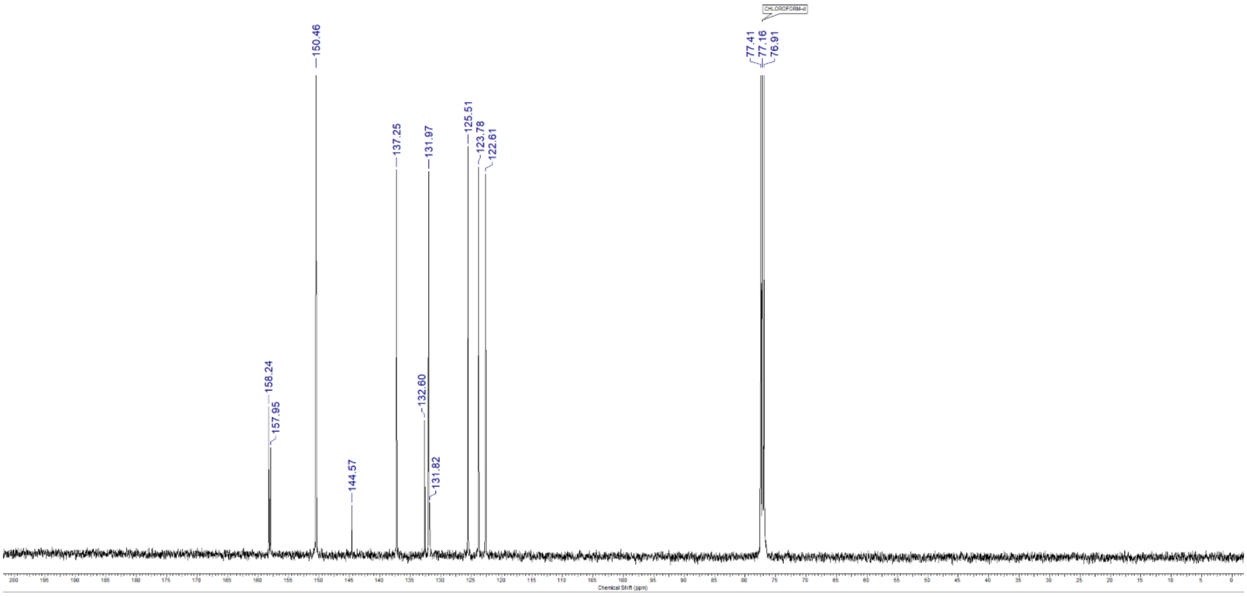

Figure S30: ^{13}C NMR spectrum of 9-(di(pyridin-2-yl)methylene)-9*H*-4,5-diazafluorene (4,5-DPDAF) in $CDCl_3$.

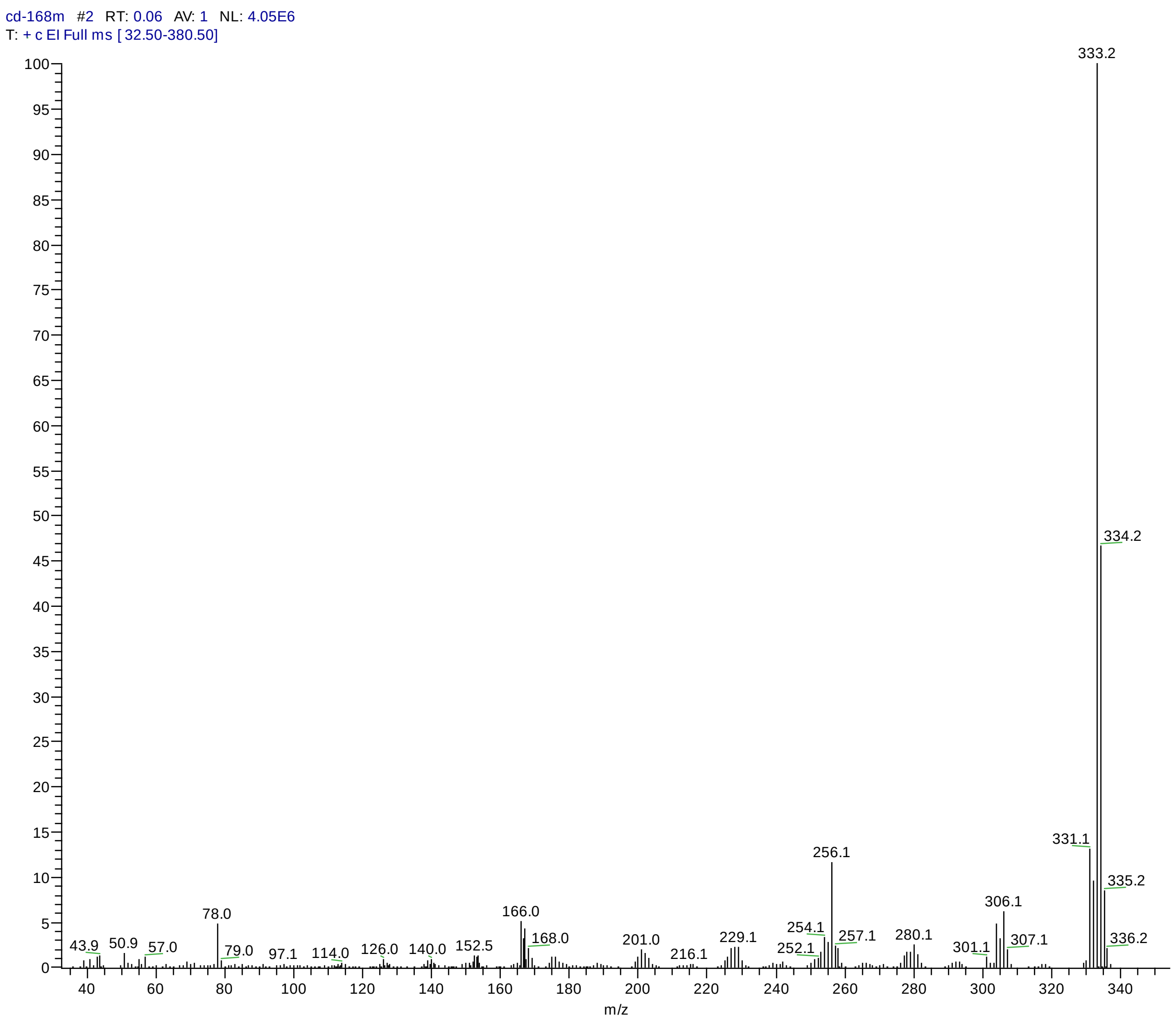

Figure S31: HRMS overview spectrum of 9-(di(pyridin-2-yl)methylene)-9*H*-4,5-diazafluorene (4,5-DPDAF) (T_{source} = 70 °C, T_{probe} = 200 °C).

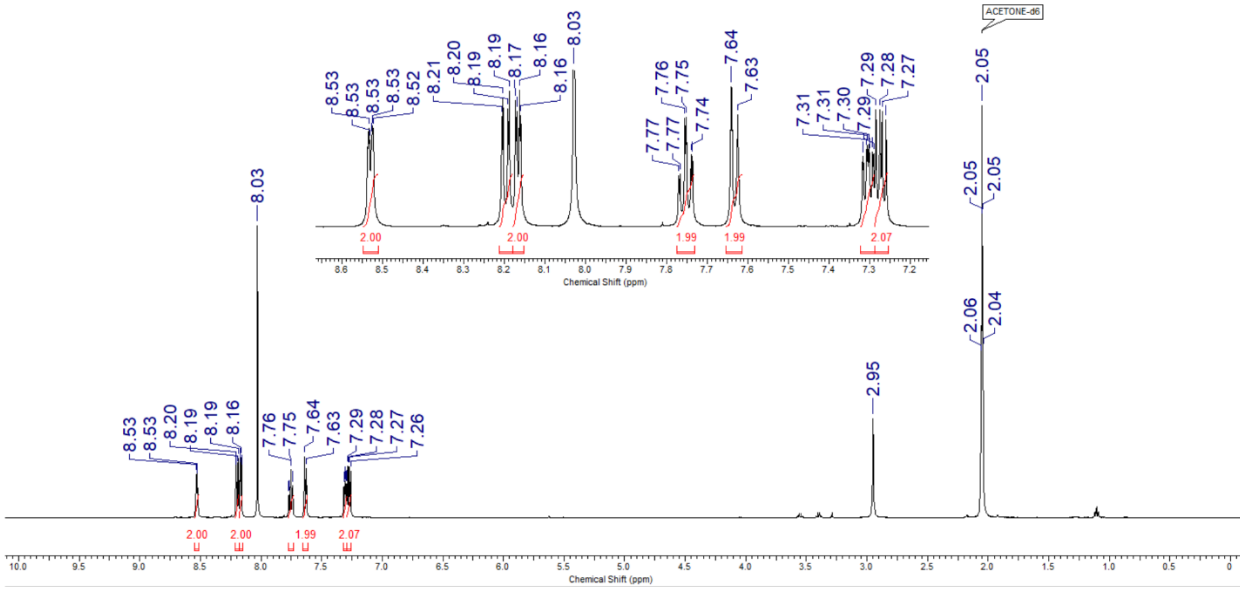

Figure S32: ^{1}H NMR spectrum of 9-(di(pyridin-2-yl)methylene)-9*H*-1,8-diazafluorene (1,8-DPDAF) in $CDCl_3$.

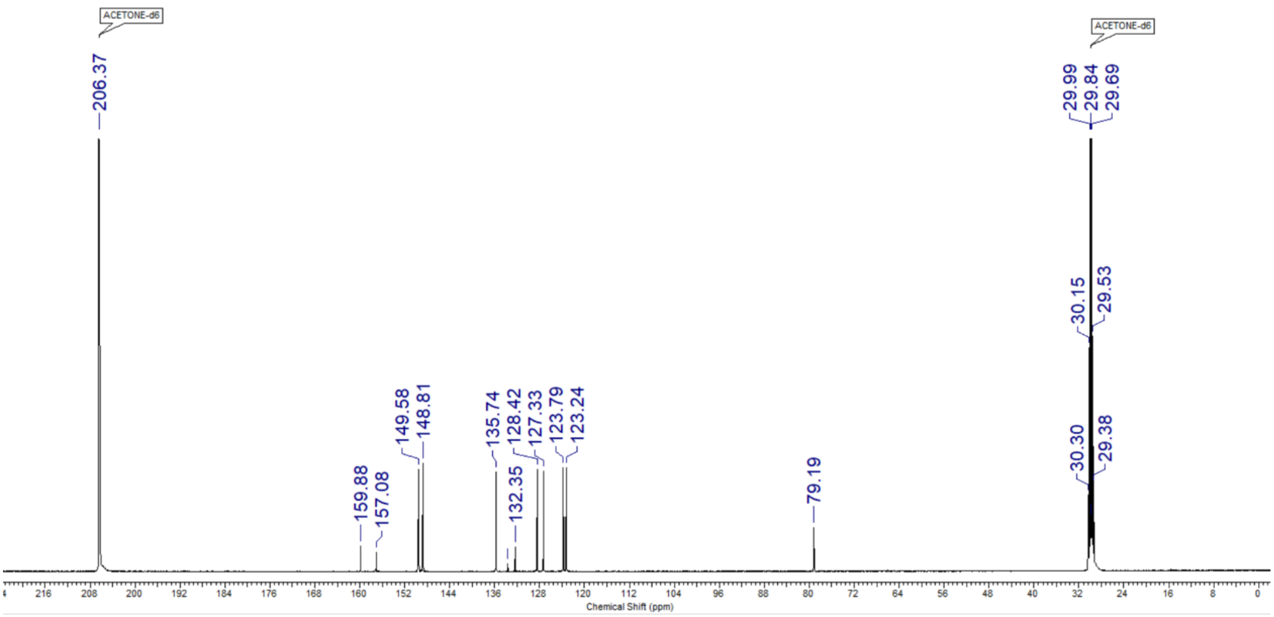

Figure S33: ^{13}C NMR spectrum of 9-(di(pyridin-2-yl)methylene)-9*H*-1,8-diazafluorene (1,8-DPDAF) in $CDCl_3$.

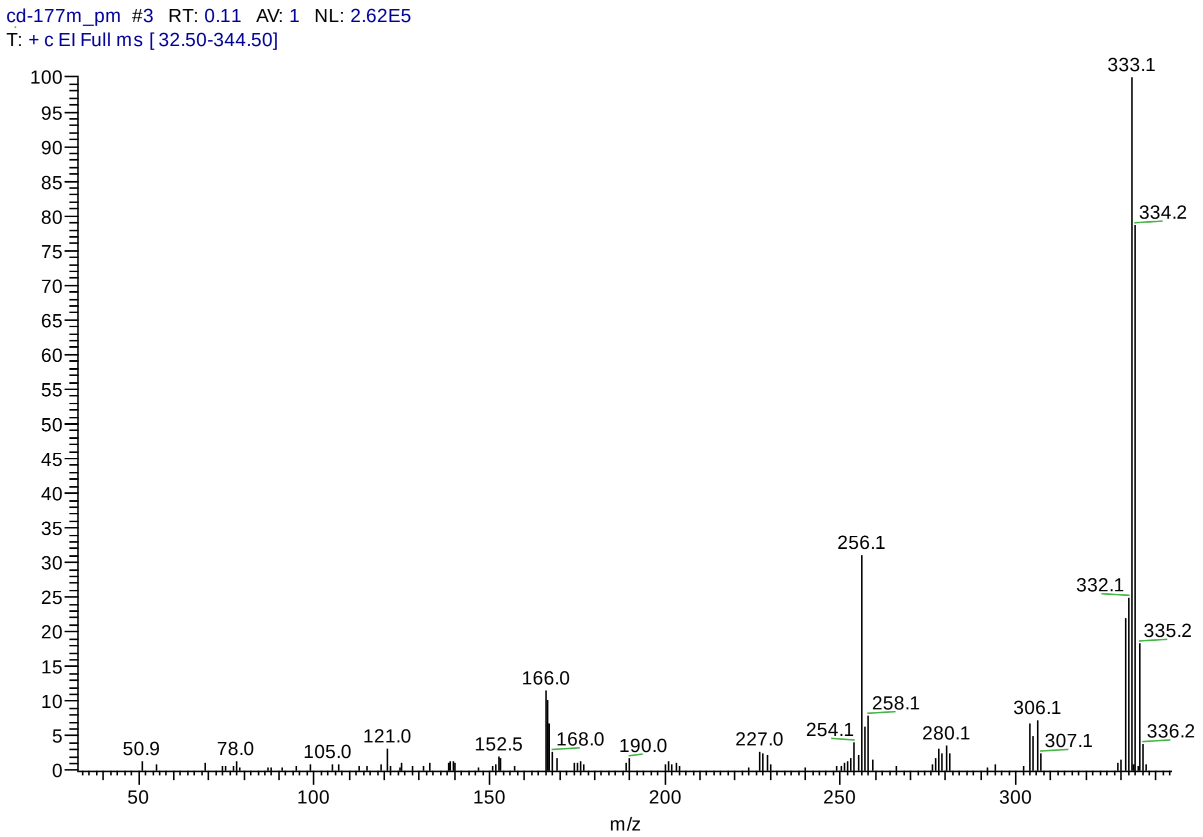

Figure S34: HRMS overview spectrum of 9-(di(pyridin-2-yl)methylene)-9*H*-1,8-diazafluorene (1,8-DPDAF) (T_{source} = 85 °C, T_{probe} = 200 °C).

Reference

1) Plater, M. J.; Kemp, S.; Lattmann, E. *J. Chem. Soc., Perkin Trans. 1* **2000,** *6*, 971–979.
